# Resolved tropical cyclones trigger CO_2_ uptake and phytoplankton bloom in an Earth system model simulation

**DOI:** 10.1073/pnas.2506103122

**Published:** 2025-12-09

**Authors:** David M. Nielsen, Fatemeh Chegini, Nuno Serra, Arjun Kumar, Nils Brüggemann, Cathy Hohenegger, Tatiana Ilyina

**Affiliations:** ^a^Climate Variability Department, Max Planck Institute for Meteorology, Hamburg 20146, Germany; ^b^Department of Earth System Sciences, University of Hamburg, Hamburg 20146, Germany; ^c^Department of Earth System Modeling, Helmholtz-Zentrum Hereon, Geestacht 21502, Germany

**Keywords:** Earth system modeling, ocean biogeochemistry, tropical cyclones, ocean carbon cycle

## Abstract

The ocean carbon cycle is directly impacted by storms in the atmosphere. Tropical cyclones (TCs), particularly, are known to drive intense air–sea CO_2_ fluxes and to trigger phytoplankton blooms. However, the current generation of Earth system models (ESM) cannot realistically represent TCs due to their coarse spatial resolution (typically 100 to 200 km grid spacing). Here, we present a km-scale coupled, global, storm- and eddy-resolving (5 km ocean, 5 km atmosphere) ESM simulation including ocean biogeochemistry that is able to resolve TCs, and the cascade of physical-biogeochemical mechanisms that unfold in their response. Our simulated TCs enhance CO_2_ fluxes by 20 to 40 times and cool the surface ocean by 2 to 3 ^°^C, thus contributing to inverting the CO_2_ flux direction from ocean outgassing to uptake. Our TCs furthermore trigger a phytoplankton bloom in autumn in the western North Atlantic, which is missed by coarser ESMs. While TCs cool the ocean surface, they also warm the subsurface, thus causing counteracting impacts on temperature-dependent organic matter remineralization. In summary, our model configuration reproduces mechanisms underlying the ocean carbon cycle variability that remained so far unresolved in ESMs. By representing fine-scale atmosphere-ocean biogeochemistry interactions in our ESM, we pave the way for future work to constrain uncertainties in the role of km-scale events in the ocean carbon cycle at global and climatic scales.

## Significance

Earth system models typically work with spatial resolution of 100 to 200 km. This is too coarse for the representation of small-scale mechanisms and extreme events, such as intense tropical cyclones (e.g. hurricanes). We present a km-scale global Earth system model simulation that resolves interactions between extremely intense cyclones and the ocean carbon cycle with unprecedentedly high resolution (5 km ocean and atmosphere). Major hurricanes locally modulate the intensity and direction of air–sea CO_2_ fluxes, while triggering phytoplankton blooms. Our modeling achievement will allow future work to reduce uncertainties in the role of km-scale processes in the ocean carbon sink and in its response to a changing climate.

The ocean absorbs about 25% of anthropogenic CO_2_ emissions every year (1, 2). However, much of this air–sea exchange of CO_2_ is mediated by processes in the surface ocean and atmosphere that are too small to be fully resolved in Earth system models (ESMs). State-of-the-art ESMs, composing the Coupled Model Intercomparison Project (CMIP6) (3) and used to inform the Intergovernmental Panel on Climate Change (IPCC), work with spatial resolution of tens to hundreds of kilometers (typically 100 to 200 km grid spacing), which is too coarse to realistically represent tropical cyclones (TCs) (4, 5). Intense TCs of category 4 to 5 in the Saffir–Simpson scale (winds >58 m/s) are particularly misrepresented, or totally absent, in coarse-resolution CMIP6-type models (4, 6). The higher-resolution subset of CMIP6 models (HighResMIP) (7), reaching up to 25 km in grid spacing, only “begin to capture some structures of TCs more realistically” (4), but biases in TC characteristics persist (8, 9). Here, we present a coupled storm- and eddy-resolving simulation (5 km ocean, 5 km atmosphere) including the ocean biogeochemistry component HAMOCC (HAMburg Ocean Carbon Cycle) (10) in the ICON (ICOsahedral Nonhydrostatic) model (11). Our model configuration has been shown to reproduce important TC characteristics, such as intensification rates, core structure (9), and interactions with ocean eddies (12). We investigate the two most *intense* TCs (i.e. category 4 or higher) in the western North Atlantic occurring in our simulation, and their impact on the ocean carbon cycle.

TCs are known to drive high CO_2_ fluxes between the ocean and atmosphere (13, 14) because CO_2_ fluxes increase with wind speed squared (15). TCs occur about as frequently in ocean outgassing regions as in uptake regions, which mitigates their global annual net effect on the ocean CO_2_ uptake (16). Nevertheless, TCs are responsible for moderating about 20 to 60% of all air–sea CO_2_ exchange in their most prevalent regions and seasons, according to mooring observations and regional modeling studies (17–20), thereby playing an important role in the ocean carbon sink variability. TCs impact the ocean CO_2_ sink for weeks after their passage by causing intense ocean heat loss, and hence cooling the surface ocean by 1 to 6 ^°^C (16, 21). Cooling decreases the ocean partial pressure of CO_2_ (*p*CO_2_) (22), thereby favoring an increasing ocean CO_2_ uptake (or decreasing CO_2_ outgassing). A realistic representation of the impact of TCs on the ocean CO_2_ sink must include both their instantaneous and long-lasting components (16).

A cascade of coupled physical-biogeochemical processes and feedbacks occur in response to TCs. First, not only do high wind speeds increase CO_2_ fluxes, they also mix the upper ocean, contributing to cooling the surface ocean, and warming the subsurface (21, 23). Ocean mixing also replenishes the sunlit upper ocean layers with nutrients and phytoplankton from the subsurface, which enhances marine net primary production in the surface ocean, as seen from satellites (24–30) and in-situ float observations (31–36). Consequently, intense TCs mediate the ocean carbon sink by enhancing the export of organic carbon to the ocean interior (37, 38). Phytoplankton blooms in the North Atlantic during autumn, particularly, are generally associated with storm-driven ocean mixing (39–41). Phytoplankton blooms also affect the penetration of shortwave radiation in the ocean, modulating their surface temperature response (e.g. refs. 42, 43, and *SI Appendix*, *The Phytoplankton-Biophysical Feedback*). Last, the cold wake of TCs is observed to negatively feedback on their own intensity and further development (44).

Investigating the impact of km-scale extreme storms on the Earth’s climate and carbon cycle remains challenging. Future projections of intense TC impacts, for example, rely on statistical or downscaling techniques (e.g. refs. 45–47), which cannot consistently simulate the feedbacks between scales (i.e. the small scales of TCs and the global and climatic scales) and neither between Earth system components (i.e. ocean–atmosphere feedbacks). Previous high-resolution modeling achievements focused on longer simulations at lower resolution (0.1^°^≈10 km) and either did not include a representation of the ocean carbon cycle (48, 49), included a simplified representation of the ocean carbon cycle with three prognostic tracers (50), or used ocean-only models forced by uncoupled atmospheric data (51). Our 5-km global ICON configuration is thus an invaluable tool that allows us to resolve coupled ocean–atmosphere interactions triggered by extreme storms within the Earth system in a consistent manner. We explore the cascade of coupled physical-biogeochemical mechanisms that unfold after the passage of the two most intense TCs occurring in the western North Atlantic in our simulations—the first intense TCs ever resolved in a fully coupled global ESM including ocean biogeochemistry. We show how such extreme storms can increase the ocean CO_2_ uptake and trigger an autumn phytoplankton bloom in the western North Atlantic.

## 1. Results and Discussion

**Fig. 1.**
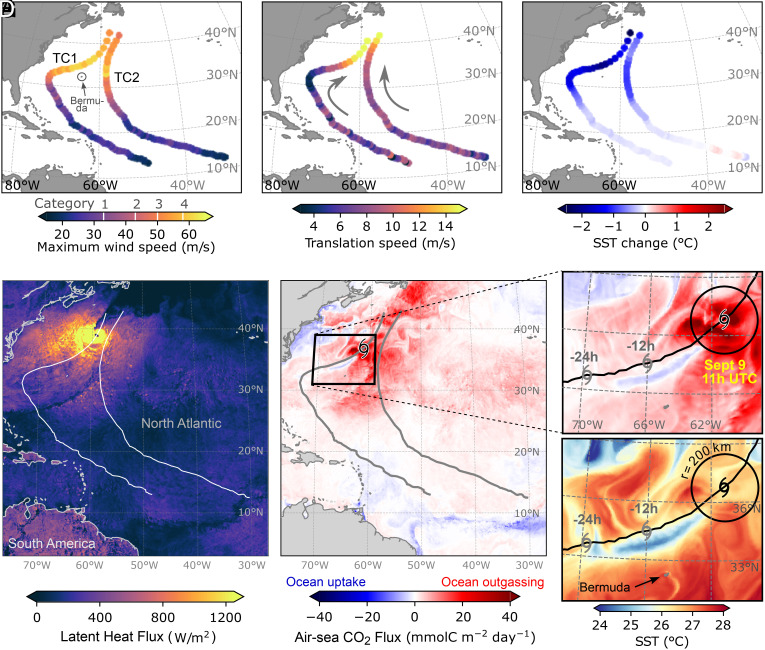
Simulated tropical cyclones characteristics. Along-track maximum surface wind speed (*A*), translation speed (*B*), and changes in sea-surface temperature (SST) due to TC passage (*C*) expressed as differences in SST from 24 h after minus 24 h before the TC averaged over a 200 km-radius circle around track points. Track points are spaced by 3 h in (*A*–*C*) for visualization. Maps of surface latent heat flux [LHF, (*D*), instantaneous] on 10 September at 16 h UTC (12-noon at the longitude of TC1 location), and air–sea CO_2_ flux [(*E*), hourly mean] at 11 h UTC (near TC1 at its maximum intensity). Both LHF and CO_2_ fluxes are positive upward (from ocean to the atmosphere), thus positive values show ocean heat loss and ocean CO_2_ outgassing. Zoomed-in views of air–sea CO_2_ fluxes (*F*) and SST (*G*), highlighting the cold wake of TC1 after its maximum intensity. Black cyclone symbols in (*E*–*G*) mark the position of TC1 at the time corresponding to the data shown, while gray cyclone symbols mark its previous positions at 12 and 24 h before.

Our simulation produces two intense TCs in the western North Atlantic. The first one (TC1) occurs between 1–10 September 2020, and the second one (TC2) occurs between 10–17 September 2020. Both TCs intensify to become hurricanes (i.e. surface wind speeds ≥33 m/s) and reach hurricane category 4 (≥58 m/s) in latitudes north of 30^°^N (Fig. 1*A*). Our two TCs follow different paths in the North Atlantic: Both TCs initially travel with a typical north-westward direction, but TC1 takes a sharp turn eastward after crossing 30^°^N while intensifying, somewhat similar to 2020 hurricane Paulette (52), but without making landfall in Bermuda. During its sharp turn, TC1 travels with low translation speeds below 6 m/s (Fig. 1*B*). On the other hand, TC2 follows a predominantly northward track after reaching hurricane intensity, and faster than TC1, with translation speeds always above 6 m/s. Slow translation speeds enable TCs more time to exert sustained wind stress on the ocean, leading to enhanced mixing and sea-surface cooling (21, 53, 54).

**Fig. 2.**
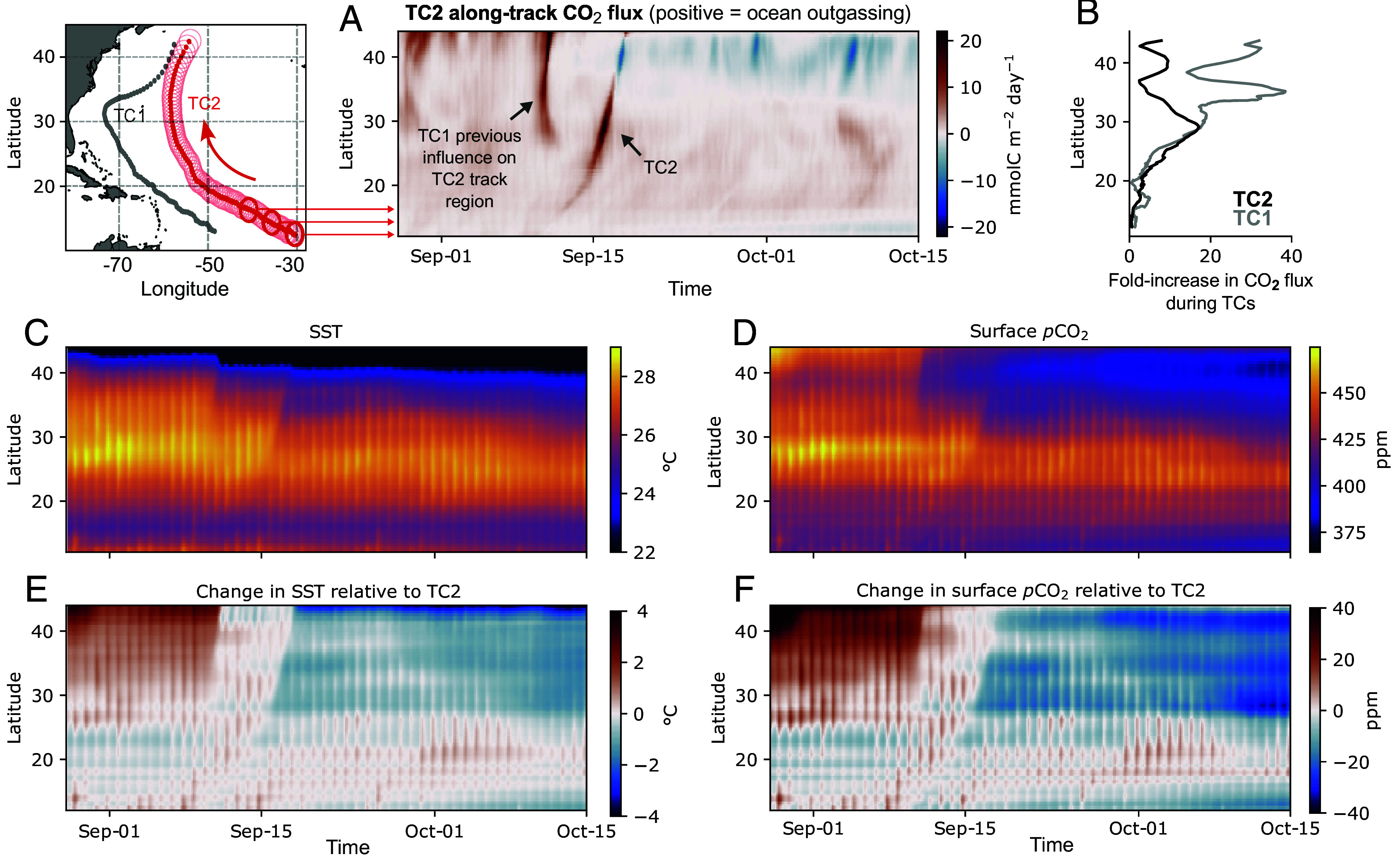
Along-track time-series diagrams of TC2 impacts on CO_2_. Time series averaged over a moving 200 km-radius circle around the center of TC2 along its track. Shown quantities are air–sea CO_2_ fluxes [(*A*), negative indicates ocean uptake], and the fold-increase in air–sea CO_2_ flux during the passage of the TCs along their tracks in comparison to 1–31 October, when no other TCs have occurred in this region (*B*). TC2 along-track absolute values of sea-surface temperature [SST, (*C*)], surface ocean pCO_2_ (*D*), and their changes with respect to 12 h before the TC2 passage [(*E* and *F*), respectively]. The vertical axes show the latitude coordinates of the center of TC2 along its track. The two-step reduction in SST and pCO_2_, one followed 5 to 6 d after the other, shows the overlapping impact of TC1 on the track of TC2, especially above 30^°^N. One can also see the daily cycle in the background as vertical stripes in all time-series diagrams. TC1 along-track time-series diagram for CO_2_ is shown in *SI Appendix*, Fig. S2.

TC1 cools the surface ocean by up to 2 ^°^C, while TC2 cools the surface ocean by about 1 ^°^C where wind speed is highest (Fig. 1*C*). The largest cumulative sea-surface cooling (2 to 3 ^°^C) takes place in the extratropics, where the two TC tracks approach each other and their areas of impact overlap (Fig. 2 *C* and *E*). This is also the warmest region along their tracks, which favors their own intensification. Sea-surface cooling is driven by the combined effect of surface heat fluxes and wind-driven vertical mixing during the TC passage, and only slightly counterbalanced by warm advection afterward (*SI Appendix*, Fig. S1; see *Ocean Tracer Budget Terms*). Horizontal advection plays a larger role in warming the wake region of TC1 after its passage, into which the Gulf Stream brings warm tropical waters.

Our results are in agreement with abundant observations showing sea-surface cooling of 1 to 6 ^°^C in response to TCs (e.g. refs. 21, 23, and 53–56). Lin et al. (54) showed that the surface cooling response to category-4 TCs, similarly to our simulated TCs, ranged in 1.1 to 2.5 ^°^C depending on TC’s translation speed. Menkes et al. (57) compiled data of over 1,000 TCs and showed that most TCs (75%) cool the surface ocean by <1 ^°^C, while only 7% of TCs cool the surface ocean by >2 ^°^C. Sea-surface cooling of ∼2.2 ^°^C and ∼3.5 ^°^C were observed in response to category-4 Hurricane Frances (23) and category-5 Hurricane Felix (53), respectively.

Our simulated TCs yield extremely high surface latent heat fluxes (LHF) of >1,200 W/m^2^ at their maximum intensity (Fig. 1*D*), higher than anywhere else on the Earth’s surface, which contributes to cooling the surface ocean. For context, the 95^th^ percentile of all year-round LHF in the western North Atlantic are as high as 100 to 350 W/m^2^ (*SI Appendix*, Fig. S2). Our results fit well within TC-driven LHF estimates from observations, ranging at 400 to 2,000 W/m^2^ compiled from aircraft and in-situ measurements (58–60). Not only do hurricanes drive extreme surface heat exchange but also intense CO_2_ exchange rates between the ocean and atmosphere.

### 1.1. Hurricanes Impact the Ocean CO_2_ Sink.

Our simulated TCs impact the ocean CO_2_ uptake in two ways. First, they drive extremely large air–sea CO_2_ fluxes *instantaneously*—during their passage—due to high wind speeds, thereby enhancing ocean outgassing (Fig. 2 *A* and *B* and *SI Appendix*, Fig. S3 *A* and *B*). These fluxes are up to 20 to 40 times larger than CO_2_ fluxes during non-TC conditions (1–31 October), in line with observations and previous regional modeling work (13, 14, 17). The largest instantaneous CO_2_ fluxes >30 mmolC m^−2^ day^−1^ occur within a radius of 150 to 200 kilometers from the TC center at their full development, where wind speeds are the strongest (Fig. 1 *E* and *F* and *SI Appendix*, Fig. S4). TC-driven CO_2_ fluxes are also well above the 95th percentile of year-round CO_2_ fluxes in this region (5 to 10 mmolC m^−2^ day^−1^, *SI Appendix*, Fig. S2). The direction of the CO_2_ flux response to TCs depends on the local imbalance in pCO_2_ between the surface ocean and atmosphere. The western North Atlantic is supersaturated in pCO_2_ before the arrival of our TCs (i.e. pCO_2_^*ocean*^ > pCO_2_^*atmosphere*^), which causes them to induce ocean CO_2_ outgassing during their passage.

Second, both TCs cause a *long-lasting* effect due to sea-surface cooling. The TC-driven rapid decrease in sea-surface temperature (SST) causes an also rapid decrease in pCO_2_ that persists for weeks after their passage (Fig. 2 *E* and *F*: changes in SST and pCO_2_ with respect to 12 h prior to TCs passage along their tracks), in line with observations and regional modeling work (e.g. refs. 13 and 61). Reducing surface pCO_2_ either decreases ocean CO_2_ outgassing or induces uptake if the pCO_2_ imbalance is inverted to undersaturated conditions (i.e. pCO_2_^*ocean*^ < pCO_2_^*atmosphere*^). The link between decreasing SST and CO_2_ flux anomalies is visible in the wake of TC1 (Fig. 1 *F* and *G*), where CO_2_ uptake is induced. The cool and low-pCO_2_ wake is shifted to the right of the TC track, which is a characteristic feature of fast-translating TCs (26, 62). The rightward shift is explained by the fact that TC wind vectors rotate in phase with the inertial current vectors to the right of the TC track (21, 63).

Sea-surface cooling plays a dominant role in decreasing surface pCO_2_ after the passage of both TCs (Fig. 2*C*–*F* and *SI Appendix*, Fig. S5*A* and *Decomposing Changes in pCO_2_*). The role of temperature is only slightly counterbalanced by the vertical diffusion of dissolved inorganic carbon (DIC), which increases surface pCO_2_ (*SI Appendix*, Fig. S5*B* and *The Role of DIC and Alkalinity*). The impact of the TCs on surface DIC through biology is negligible compared to that of vertical diffusion by turbulent mixing (*SI Appendix*, Fig. S8 and *The Impact of Biology on Surface pCO_2_*). Increasing surface salinity also contributes to increasing surface pCO_2_, especially at latitudes north of 35^°^N (*SI Appendix*, Fig. S5*C*), where the vertical diffusion of salinity dominates over freshening due to surface fluxes (precipitation > evaporation during both TCs). The relative role of temperature, DIC, and salinity in changing surface pCO_2_ in response to TCs varies substantially in different regions and between individual TC episodes (e.g. refs. 17, 18–20, 61, and 64). In the North Carolina estuary, for example, TCs have been observed to cause net CO_2_ outgassing, where increasing surface DIC outweighs the cooling effect (65–67). Preexisting gradients in temperature, DIC, alkalinity and salinity, as well as cyclone intensity and translation speed, and thus their ability to induce vertical mixing, will altogether determine their net effect on pCO_2_ anomalies (68, 69).

TC-driven sea-surface cooling reduces surface ocean pCO_2_ by ∼10 to 40 ppm to undersaturated conditions in the extratropics (Fig. 2*F* and *SI Appendix*, Fig. S4*A*). Consequently, both TCs contribute to inverting the direction of CO_2_ fluxes from ocean outgassing to uptake (Fig. 2*A* and *SI Appendix*, Fig. S2*A*).

Accumulating CO_2_ fluxes from 24 h preceding the TCs reveals that the long-lasting cooling effect outweighs the instantaneous outgassing by mid September, especially after TC2 and at latitudes higher than 30^°^N, resulting in a net increase in ocean CO_2_ uptake (*SI Appendix*, Fig. S2*C*). Considering only their instantaneous effect, the two TCs together induce about 1.25 TgC in CO_2_ outgassing (1 TgC = 10^12^g or 1 million metric tons of carbon) during their passage (integrated over a 200 km-radius circle along their tracks during their life time, i.e. 1–17 September). This is equivalent to ∼26% of the outgassing that took place in their track region in the warm season (July 1st–September 31st: +4.8 TgC), while also equivalent to less than 2% of the CO_2_ uptake that takes place in the same region over the rest of the year (−63.8 TgC).

While it is straightforward to quantify the *instantaneous* outgassing impact of our TCs by integrating CO_2_ fluxes in their track region during their passage (i.e. their life time), quantifying their *long-lasting* cooling impact on the CO_2_ uptake is less objective. Quantifying the long-lasting impact of TCs on CO_2_ uptake is sensitive to the subjective choice of an integration time window. Here, instead, we underline that our TCs contribute to inverting the air–sea CO_2_ flux—from ocean CO_2_ outgassing to uptake—by modulating surface pCO_2_ during their highest intensity as category-4 hurricanes. We thereby represent variability in the ocean carbon cycle that has remained so far unresolved in global coupled ESMs.

Lévy et al. (16) showed that the opposing instantaneous and long-lasting TC impacts on air–sea CO_2_ fluxes nearly cancel out each other at the global scale, after analyzing hundreds of TCs with a coarser global ocean model (0.5^°^ to 2^°^ horizontal resolution) forced by atmospheric reanalyses. Regionally, however, the impact of TCs on the annual net air–sea CO_2_ flux varies: In the western North Atlantic, eastern North Pacific, and western North Pacific, TCs would account for about 1%, 2%, and 33% of the annual net CO_2_ exchange, respectively (16). In the Southeast China Sea, observations suggested that TCs account for 20 to 60% of the net CO_2_ uptake during summer (18–20).

### 1.2. Hurricanes Trigger Second Phytoplankton Bloom in Autumn.

**Fig. 3.**
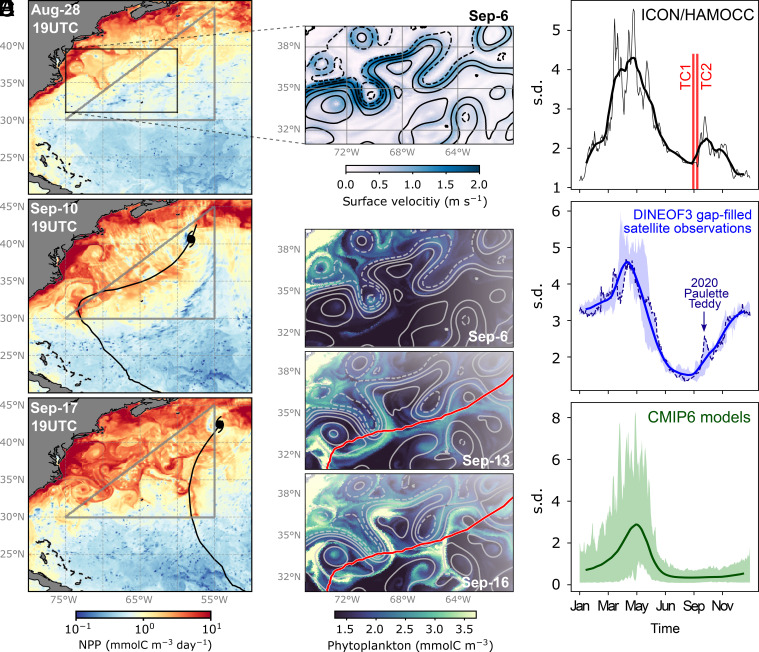
Growth and spread of phytoplankton in response to TCs. (*A*) Vertically averaged net primary production (NPP) on 28 August 2020 (*Upper* panel, before the hurricanes), on 10 September 2020 (*Middle* panel, after TC1), and on 17 September (*Lower* panel, after TC2). (*B*) Zoomed-in map of surface velocities and sea-surface height anomalies (contours) in the Gulf Stream box shown in (*A*). (*C*) Temporal evolution of vertically averaged phytoplankton concentrations in the Gulf Stream box on 6 September (*Upper* panel, before TC1), on 13 September (*Middle* panel), and 16 September (*Lower* panel) after TC1, whose track is shown in red. All data in (*A*–*D*) are generated by our ICON/HAMOCC simulation. (*D*) Time series of near-surface Chlorophyll-a concentrations averaged over the western North Atlantic [triangle in (*A*)] in our 1-y ICON simulation, (*E*) from satellite observations in 2018–2022, and (*F*) in a CMIP6 multimodel mean in 2010–2014. In (*E*), the shade shows the full range, the thick line shows the mean, and the dashed line highlights year 2020, when hurricanes Paulette and Teddy took place. Chlorophyll-a data in E are obtained from NOAA CoastWatch at coastwatch.noaa.gov/cwn/index.html, which are derived from multiple satellites (VIIRS-SNPP, VIIRS-NOAA-20, and OLCI-Sentinel-3A) and processed with the DINEOF gap-filling method (70). CMIP6 models with daily chlorophyll data available are MPI-ESM-LR (HAMOCC), IPSL-CM6A-LR (NEMO-PISCES), and CESM2 (MARBL). In (*F*), the shade shows the full model range and the line shows the multimodel mean. Thin lines and shades in (*D*–*F*) show daily data, while thick lines show a 31-d running mean. Units in (*D*–*F*) are SDs of the respective time series.

We simulate a local increase in net primary production (NPP) by at least one order of magnitude in response to both TCs (Fig. 3*A*). The magnitude of the simulated NPP response is in agreement with satellite and in-situ observations (e.g. refs. 24, 25, 29, 31, and 33). Similarly to the cooling effect, the NPP increase is also shifted to the right of the TC tracks, according to theory and observations (21, 71), which is especially true for fast-translating TCs (24, 54). However, the TC-driven increase in phytoplankton concentration is not spatially restricted to the vicinity of their tracks. The Gulf Stream and its mesoscale meanders play an important role in transporting phytoplankton away from the track of TC1 after its passage (Fig. 3 *B* and *C*). While the increase in phytoplankton concentrations is mainly produced locally within the TC track (*SI Appendix*, Fig. S9 *A* and *B*), their spatial spread is mainly done by geostrophic currents, which are characterized by flowing along contours of sea-surface height anomalies. That is, the increase in phytoplankton concentration in the wake of TCs is thus partly counterbalanced by horizontal advection (*SI Appendix*, Fig. S9*C*). Consequently, our simulated TCs trigger a phytoplankton bloom that spreads across the western North Atlantic beyond the region of hurricane-force winds. This highlights the role of the pronounced mesoscale, high eddy kinetic energy currents in advecting phytoplankton horizontally away from the TC tracks, which continues for several days after their passage. In fact, TCs themselves also intensify surface currents (72, 73), contributing to further spread their biological impact.

The simulated TCs contribute to the development of a phytoplankton bloom in autumn, distinct from the spring bloom (Fig. 3*D*). The observed seasonal cycle of chlorophyll-a in the western North Atlantic shows a peak in spring, a minimum by the end of summer, and a growing phase during autumn (Fig. 3*E*). Although our simulation does not yet perfectly reproduce the observed phytoplankton phenology, it represents an improvement compared to state-of-the-art ESMs, in that our TCs trigger a phytoplankton bloom in autumn. ESMs are able to represent the spring bloom from observations, but they generally fail to capture the autumn recovery (Fig. 3*F*). A realistic representation of phytoplankton phenology by ESMs has been a long-lasting challenge, especially in capturing such secondary blooms in mid latitudes (74).

Autumn phytoplankton blooms are observed along the North Atlantic current further north at 40 to 50^°^N (39), generally triggered by mixed layer deepening by storms, not necessarily only TCs (40, 75, 76). Nevertheless, the role of TCs in triggering autumn blooms has been repeatedly reported in observations (e.g. refs. 24, 35, 54, and 77).

The contribution of TCs to the global biological carbon drawdown has been shown to be small (about 1%, refs. 27 and 57). Not all TCs necessarily trigger phytoplankton blooms (30, 54), and some TCs only redistribute phytoplankton vertically, without causing an increase in biomass (36). However, TCs contribute significantly to annually integrated NPP at regional scales: 5 to 10%, 10 to 15%, and up to 20% in the western North Atlantic, eastern North Pacific, and western North Pacific, respectively (57). In our simulations, integrating NPP in September–October yields 16 gC/m^2^ in the western North Atlantic (triangular region in Fig. 3*A*), which corresponds to 12% of the region’s total annual NPP (128 gC/m^2^). This is only an upper bound for the TCs’ contribution, as naturally not all NPP in October–November is triggered by the cyclones.

Despite small fractions of the annual total, TCs have been shown to drive interannual variability of chlorophyll-a in the western North Atlantic by modulating the mixed-layer depth (26, 40). In 2020, hurricanes Paulette and Teddy followed paths similar to those of our simulated TCs, and triggered a September peak in phytoplankton concentrations in the western North Atlantic (Fig. 3*E* and *SI Appendix*, Fig. S10). By resolving the km-scale interactions between atmosphere and ocean biogeochemistry, we also increase the realism of the biological response to intense TCs.

### 1.3. Cascading Physical-Biogeochemical Response to Hurricanes.

**Fig. 4.**
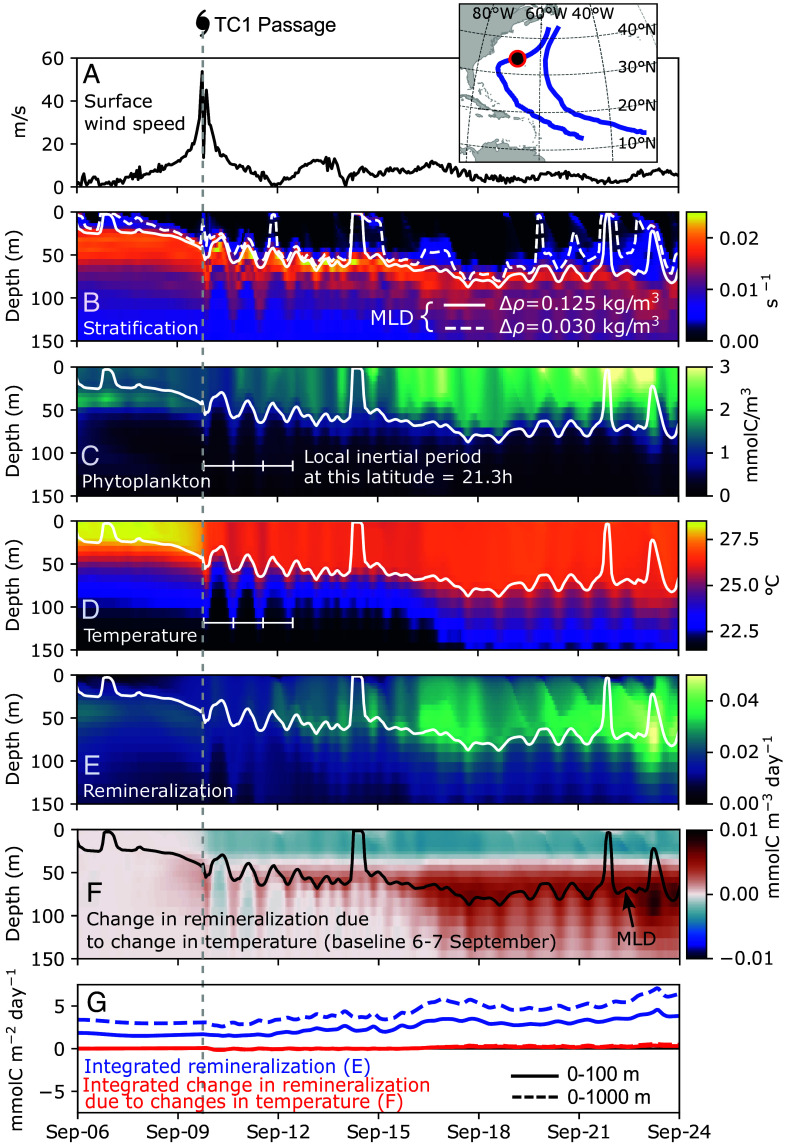
Cascading physical and biogeochemical response to hurricane passage. Time series from a fixed point in space (about 34^°^N and 67^°^W; see *Inset* map) on the track of TC1, depicting conditions from before and after its passage as a category 4 hurricane. Variables shown are surface wind speed (*A*), and vertical profiles of density stratification expressed as the Brunt-Väisälä frequency (*B*), phytoplankton concentration (*C*), temperature (*D*), aerobic organic matter remineralization (*E*), and changes in remineralization due to changes in temperature (*F*). Profiles (*E* and *F*) are vertically integrated and shown in panel (*G*) in blue and red lines, respectively, from the surface until 100 m (solid lines) and 1,000 m (dashed lines). Small white axes illustrate the local inertial period at this latitude (21.3 h). Contours in (*B*–*F*) show the mixed-layer depth, defined as the depth at which density becomes 0.03 kg/m^3^ (dashed) and 0.125 kg/m^3^ (solid lines) greater than at the surface.

The TCs decrease upper-ocean stratification, thereby increasing the mixed-layer depth by 20 to 40 m at their maximum intensity (Fig. 4 *A* and *B* and *SI Appendix*, Fig. S11*A*). Mixed-layer depth starts increasing already before the TC arrives, which follows the increase in surface wind speeds from below 5 m/s to above 50 m/s in about two days’ time. Mooring and ship observations show that hurricanes Nicole, Frances, and Felix (categories 3, 4, and 5, respectively) deepened the mixed layer by up to about 25 m (37), 80 m (23), and 25 m (78), respectively, during their passage in the North Atlantic. Variability in mixed-layer response depends not only on TC intensity but also on preexisting conditions (e.g. pre-TC mixed-layer depth), TC’s translation speed, the presence of mesoscale eddies (12, 21, 54).

Strong surface wind speeds also induce a pulse of nutrients into the euphotic zone by turbulent mixing (*SI Appendix*, Fig. S12 *A*–*C*). While NPP responds immediately to the presence of nutrients (Fig. 3*A*), maxima in phytoplankton concentration occur and persist from days up to 2 to 3 wk afterward (Fig. 4*C* and *SI Appendix*, Fig. S9*A*) in line with previous observations and modeling work (35, 57, 79). Phytoplankton is continuously diffused from the upper layers downward by wind-driven mixing (*SI Appendix*, Figs. S12*E* and S13 *A* and *B*). The vertical diffusion and advection of phytoplankton is mostly contained within the upper 100 m in the wake of TCs, that is, integrated changes in phytoplankton biomass along the TC tracks due to vertical diffusion and vertical advection are negligible (*SI Appendix*, Fig. S9 *D* and *E*) compared to horizontal and source terms (*SI Appendix*, Fig. S9 *B* and *C*).

Near-inertial oscillations are triggered by the TCs, and become the prevailing mode of variability in the upper ocean in their wake region, propagating horizontally and vertically downward (21). The horizontal advection of phytoplankton takes place mostly at near-inertial frequencies (*SI Appendix*, Figs. S12*D* and S13*C*), which underscores their role in spatially spreading the TC-driven bloom. The downward propagation and breaking of near-inertial waves also transport kinetic energy (71) and further mix the thermocline, thus contributing to sustaining subsurface warming for weeks after the TC passage, when they break and dissipate energy away from their origin (Fig. 4*D*) (80, 81). The surface cooling and subsurface warming—a typical dichotomy in the wake of TCs (21, 82)—is reproduced in our simulations, where 50 m-temperatures increase by 1 to 1.5 ^°^C in the wake of TCs (*SI Appendix*, Fig. S11*B*).

Our simulated TC-driven subsurface warming contributes to enhance organic matter remineralization (Fig. 4*E*), in addition to the increase in biological production itself. We compute changes in remineralization due to temperature changes alone by recalculating remineralization while fixing temperatures to pre-TC conditions (Fig. 4*F* and *SI Appendix*, *Organic Matter and Remineralization*). Subsurface warming causes an increase in remineralization below ∼50 m, which corresponds to the pre-TC mixed-layer depth at this point. On the other hand, remineralization decreases in the layers above, between the surface ocean until ∼50 m, due to cooling. Consequently, due to such competing effects, vertically integrated changes in remineralization due to changes in temperature are negligible (Fig. 4*G*).

Similar to the increase in NPP, the TCs cause an increase in organic matter export to depth (*SI Appendix*, Fig. S14*A*), in agreement with observations (37, 38). The ratio between integrated NPP and export (i.e. the export ratio) does not change substantially in response to the TCs, especially since integrated changes in remineralization due to temperature changes are negligible (*SI Appendix*, *Organic Matter Export*). Considering a lag of about 9 d between integrated NPP and export at 90 m, export ratios remain at about 8 to 12% in the western North Atlantic throughout the year in our simulation (*SI Appendix*, Fig. S14*B*). Our export ratios compare well with observations-based estimates near Bermuda [6.3 to 8.4% at 150 m (83)]. In the western North Atlantic, export ratio estimates range at least between 5 to 30%, with values generally increasing from the open ocean toward coastal zones (see refs. 84, 85 and references therein).

In the beginning of their trajectory, especially south of 25^°^N, the TCs are not intense enough to break stratification (*SI Appendix*, Fig. S15 *A* and *B*). On the other hand, in latitudes north of 30^°^N, their effect is compound. After the passage of TC1 at about 34^°^N on September 10th, we see a further increase in mixed-layer depth by about 20 to 30 m (Fig. 4). This is explained by, first, the passage of an extratropical storm on September 13th and, second, the indirect impact of TC2 from afar on September 17th, whose trajectory approaches that of TC1. Interactions between impacts of TC1 and TC2 are more clearly visible further north, when their trajectories are less than 300 km apart (*SI Appendix*, Fig. S15 *C* and *D*), allowing for an overlap. At the same time, winds as high as 16 to 24 m/s occur 200 to 300 km from the TC centers in latitudes north of 35^°^N (*SI Appendix*, Fig. S4). Near-inertial waves generated by TC1 are broken, or dimmed by destructive interference, by the interactions with the following storms, which contributes to further ocean mixing, and to the consequent cascade of biogeochemical effects (Fig. 4 and *SI Appendix*, Fig. S15 *C* and *D*).

## 2. Conclusion

We conducted the first storm- and eddy-resolving (5 km ocean, 5 km atmosphere) global coupled ESM simulation including ocean biogeochemistry, which allowed us to resolve atmosphere-ocean biogeochemistry interactions in the km-scale within the Earth system. We investigated two particularly intense TCs reaching category-4 hurricane force (surface wind speed >58 m/s) in the North Atlantic, which state-of-the-art ESMs have been, so far, unable to capture.

We resolved the cascade of physical-biogeochemical effects that unfold in response to TCs in a global coupled ESM. Extreme wind speeds enhance CO_2_ outgassing by 20 to 40 times from the western North Atlantic, initially supersaturated in pCO_2_. The TCs also cool the surface ocean by 1 to 2 ^°^C each, decreasing the surface ocean pCO_2_ by 10 to 40 ppm to undersaturated levels. Vertical mixing of DIC only slightly counteracts the cooling effect on surface pCO_2_. Biology plays a minor role in modulating surface pCO_2_ in comparison to temperature and mixing effects. Consequently, the simulated TCs contribute to inverting the CO_2_ flux direction from ocean outgassing to ocean uptake. Moreover, the intense TCs increase NPP locally by at least a factor of 10, triggering a phytoplankton bloom in autumn, which typical ESMs so far fail to represent. This distinct autumn bloom persists in the western North Atlantic for weeks after the TC passage. Phytoplankton is spread horizontally by ocean mesoscale currents, especially in the vicinity of the Gulf Stream. The TCs also change organic matter remineralization (by up to ∼20%) via temperature changes, but compensating effects (i.e. surface cooling and subsurface warming) nearly cancel out each other, thus causing a minor net impact on the export-to-NPP ratio. The proportional increase in NPP and export highlights a potential role for TCs in promoting long-term carbon storage in the ocean depth.

The frequency and intensity of extreme weather events increase in response to climate change. The proportion of intense TCs is projected to increase in the future with *“high confidence”* according to the latest IPCC report (4), and so is their impact on the ocean carbon cycle. TCs intensify particularly fast in response to marine heatwaves (86, 87), which will also tend to become more intense in the future (88, 89). The large variability between TCs and extreme events overall prevents generalizations of our findings in time or space, for which longer simulations are needed. Longer km-scale ESM simulations (with grid spacing of 5 km or finer) are necessary to realistically represent such future changes, their impacts, and feedbacks to climate by modulating the ocean CO_2_ sink. Our work shows that this is now possible. By resolving small-scale interactions between atmosphere and ocean biogeochemistry in a global coupled ESM, we bridge the gap between km-scale mechanisms and the ocean carbon cycle at global and climatic scales.

## 3. Materials and Methods

### 3.1. ICON Model and Simulations.

We use the ICOsahedral Nonhydrostatic (ICON) coupled model in the “Sapphire” configuration (11), which includes atmospheric, ocean, land, and ocean biogeochemistry components. ICON uses an unstructured triangular grid, here configured with nominal mean horizontal resolution of 5 km in all components. The atmospheric dynamics are based on the numerical weather prediction (NWP) model by the German Weather Service (DWD) (90). In the atmosphere, convection is explicitly resolved, while parameterizations are only used in processes that cannot be resolved in the km-scale, namely radiation, microphysics, and turbulence (11). The atmosphere is coupled to the ocean and sea-ice model ICON-O (91). Here, ICON-O is configured with 72 layers in z* coordinates, which distributes sea-surface height anomalies at every time step in all model layers. In our configuration, ICON-O resolves ocean eddies explicitly, and therefore parameterizations for eddy-induced horizontal diffusion and advection are switched off [i.e. GM-Redi (92, 93)]. Parameterizations maintained include a turbulent kinetic energy (TKE) scheme for vertical turbulent mixing, and a biharmonic velocity dissipation scheme (91). We refer the reader to Korn et al. (91) for more details on model parameterizations. Land biogeophysics and biogeochemistry are represented in JSBACH (94) version 4. Ocean biogeochemistry is included with the HAMburg Ocean Carbon Cycle (HAMOCC) model (10).

HAMOCC simulates biogeochemical processes in the water column, including interactions with the atmosphere and sediment. HAMOCC was initially developed based on a nutrient-phytoplankton-zooplankton-detritus (NPDZ) framework (95) but is now expanded to include the representation of 20 biogeochemical tracers (10). Marine biology in HAMOCC is represented by two tracer variables: bulk phytoplankton, which includes calcifying and silicifying species, and cyanobacteria (diazotrophs) (96). Phytoplankton growth is colimited by nitrate, phosphate, and iron, while cyanobacteria can utilize dinitrogen. Our HAMOCC configuration includes the representation of dynamic particle sinking speeds in the water column through the M4AGO scheme (84), and temperature-dependent organic matter remineralization. A constant carbon-to-nutrient ratio is assumed for all marine organic matter following a global Redfield ratio concept (97). HAMOCC is embedded in ICON-O, and therefore they share the same grid configuration.

The air–sea CO_2_ flux is calculated in HAMOCC as $F=k_{w}\;S_{CO_{2}}$$\left(p{\mathrm{CO}}_{2}^{\mathrm{ocean}}-p{\mathrm{CO}}_{2}^{\mathrm{atmo}}\right)$, where $k_{w}$ is the gas transfer velocity, a function of temperature and the squared 10 m wind speed, calculated according to ref. 15, $S_{CO_{2}}$ is the solubility of CO_2_ in seawater, a function of temperature, calculated according to ref. 98, $p{\mathrm{CO}}_{2}^{\mathrm{ocean}}$ is the surface ocean pCO_2_ calculated at each time step from DIC and alkalinity concentrations, and $p{\mathrm{CO}}_{2}^{\mathrm{atmo}}$ is the surface atmospheric pCO_2_ corrected for the total atmospheric pressure following the protocols for the Ocean Model Intercomparison Project (OMIP) (99). In our simulation, atmospheric CO_2_ concentrations are uniformly prescribed over the entire ocean surface following year 2020 values, and do not change in response to air–sea CO_2_ fluxes.

Our coupled ICON configuration (ocean and atmosphere physics) is the same as that from Baker et al. (9), where they show that key TC characteristics are simulated realistically compared to observations, namely TC intensification rates, TC inner-core structure, and the relationship between TC size and intensification. Our ICON configuration only differs from that of Baker et al. in that we include the representation of ocean biogeochemistry with HAMOCC, and therefore also the feedback to the ocean physics through the dynamic representation of sunlight absorption by phytoplankton (100).

**Fig. 5.**
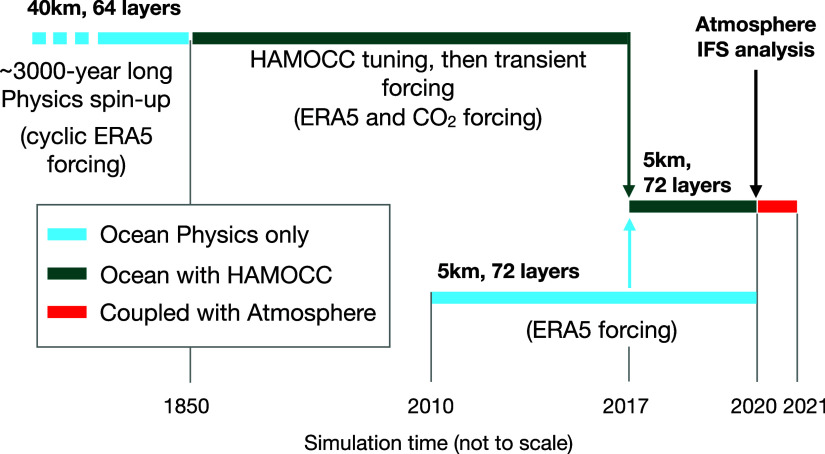
Step-wise strategy for initialization. Schematics of previous simulations performed for tuning and spin-up, leading to the initial conditions used for our coupled simulation.

We use a step-wise strategy of model complexity and resolution to arrive at the initial conditions for our coupled model simulation. This strategy consists in initializing our model from previously tuned and spun-up simulations at coarser resolutions (Fig. 5), providing enough time for the next simulation to adapt to the given initial conditions. Our coupled simulation starts from a transient ocean 5 km ICON-O/HAMOCC simulation, and from the Integrated Forecast System (IFS) atmospheric analysis at 01-01-2020 (101). From this date onward, our model “runs freely.” That is, our coupled simulation does not assimilate observed data and, due to the chaotic nature of the atmosphere, it is not expected to reproduce the exact weather systems that occurred in reality in that year. The ocean biogeochemistry is taken from a well-tuned and well spun-up coarser ICON-O/HAMOCC simulation (40 km spatial resolution, 64 vertical layers) which, in turn, follows from a >3,000 y-long ICON-O physics-only spin-up. The high-resolution ocean physics is taken from an ICON-O 5 km transient simulation started in 2010 forced with the ERA5 reanalysis (102) and increasing atmospheric CO_2_ concentrations. We remap the 40 km HAMOCC variables onto the 5 km ICON-O grid and run ICON-O with HAMOCC at 5 km from 01-01-2017 for three years to allow for enough time for the biogeochemical tracers in the upper ocean layers to adapt to the ocean mesoscale field. Spinning-up HAMOCC to reach quasi-stability in all layers and biogeochemical tracers, including in the ocean sediment, requires hundreds or thousands of years of simulations. Such long spin-ups are still impractical at 5 km resolution. The here-applied cascading strategy allows us to generate initial conditions with relatively stable conditions (sufficiently small drifts) in the euphotic zone—where we focus on in this study—and that are consistent with the ocean physics from the 5 km ocean model. Moreover, any long-term trends are small relative to the variability in our short 1-y simulation. After running the coupled model for one year with daily output, we repeat the simulation from 20-August to 31-October with hourly output.

### 3.2. TC Tracking.

We deploy a tracking algorithm that only requires 2D surface fields and closely follows the approach taken by Shimura et al. (103). The algorithm takes the hourly mean sea-level pressure (MSLP) and near-surface wind speed over both land and ocean as inputs and consists of two main steps: 1) identifying candidate tropical cyclone points and 2) stitching together the points to create tracks. For each time step, we begin by finding the grid cell with the lowest MSLP. If the MSLP is less than 1,010 hPa, the grid cell is stored as a candidate TC point and all grid cells within an 800 km radius are masked. This is repeated until the MSLP of all remaining grid cells exceeds 1,010 hPa. In the second step, the tracks are formed by connecting candidate points with a surface wind speed of at least 17 m/s that are sufficiently close in time and space. To this end, we start by selecting the candidate point at the earliest possible time step within the region 0^°^ N to 35^°^ to mark the start of a track. We then look for the next candidate point to add to the track, which must lie within 100 km and the next 24 h. Priority is given to points closer in time. In the case where multiple points are found for the same time step, the one with the lowest MSLP is selected and the remaining points are removed. The track is successively extended until no more candidate points satisfy the above conditions. This process is repeated until all candidate points are added to tracks or removed. In a further postprocessing step, we retain only those tracks with a lifetime greater than 24 h and lifetime maximum intensity that exceeds hurricane strength (>33 m/s).

Our algorithm identifies 42 TCs that reach at least category-1 hurricane intensity in our 1-y global simulation. We do not consider less intense tropical storms and depressions. Three of the 42 hurricane-force TCs take place in the North Atlantic. Here, we focus on the two *intense* TCs (i.e. category 4 or higher), since they are those typically missed by ESMs. A third and weaker TC occurs in the Gulf of Mexico, reaching hurricane category 1 shortly before making landfall.

### 3.3. Ocean Tracer Budget Terms.

We explicitly calculate the contribution of each term composing the tendencies of most biogeochemical tracers, temperature, and salinity, during the ICON model integration. This tendency decomposition is calculated online at each time step and stored as daily or hourly means, depending on the output frequency. The total tendency of tracer *C* is calculated as

[1] $$\begin{array}{cc} \frac{\partial C}{\partial t}= & -U\cdot \nabla C+\frac{\partial }{\partial z}\left(k_{v}\frac{\partial C}{\partial z}\right)\\ & +\text{Surface fluxes}\\ & +\;\text{Biogeochemical sources and sinks}, \end{array}$$

where the terms on the right-hand-side are the contributions from advection (we calculate horizontal and vertical advection separately), vertical diffusion by turbulent mixing, and surface fluxes, respectively. U=(u,v,w) is the ocean velocity vector, and $k_{v}$ is the vertical diffusion coefficient obtained using a turbulent kinetic energy (TKE) closure parameterization (see ref. 91).

### 3.4. Decomposing Changes in pCO_2_.

Following Takahashi et al. (22), we decompose the total change in surface pCO_2_ triggered by the passage of the TCs between contributions from changes in sea-surface salinity (SSS), sea-surface temperature (SST) and surface DIC and alkalinity concentrations as $\delta pCO_{2}^{\mathrm{Total}}=\delta pCO_{2}^{\mathrm{SST}}+\delta pCO_{2}^{\mathrm{SSS}}+\delta pCO_{2}^{\mathrm{DIC}}+\delta pCO_{2}^{\mathrm{Alk}}$. Here, “*δ*” denotes changes in time with respect to the TC passage, not to be confused with $\Delta p{\mathrm{CO}}_{2}$, which commonly represents the pCO_2_ imbalance between ocean and atmosphere. Each term is calculated by scaling the simulated surface pCO_2_ with changes in SST, SSS, DIC, and alkalinity:

[2] $$\begin{array}{cc} \delta pCO_{2}^{\mathrm{SST}} & =\gamma_{\mathrm{SST}}\;pCO_{2\;\mathrm{ref}}\;\delta SST\\ \delta pCO_{2}^{\mathrm{SSS}} & =\gamma_{\mathrm{SSS}}\;pCO_{2\;\mathrm{ref}}\;\delta SSS/SSS_{\mathrm{ref}}\\ \delta pCO_{2}^{\mathrm{DIC}} & =\gamma_{\mathrm{DIC}}\;pCO_{2\;\mathrm{ref}}\;\delta DIC/DIC_{\mathrm{ref}}\\ \delta pCO_{2}^{\mathrm{Alk}} & =\gamma_{\mathrm{Alk}}\;pCO_{2\;\mathrm{ref}}\;\delta Alk/Alk_{\mathrm{ref}}, \end{array}$$

where “*ref*” denotes a local reference value taken 24 h prior to the TC passage, $\gamma_{\mathrm{SST}}=0.0423$ ^°^C^−1^, $\gamma_{\mathrm{SSS}}=1$ and, from our model data, we use the approximation presented by ref. 104 to calculate:

[3] $$\begin{array}{cc} \gamma_{\mathrm{DIC}} & =\frac{3\;Alk\;DIC-2\;DIC^{2}}{\left(2\;DIC-Alk)(Alk-DIC\right)}\\ \gamma_{\mathrm{Alk}} & =-\frac{Alk^{2}}{\left(2\;DIC-Alk)(Alk-DIC\right)} \end{array}$$

The coefficient $\gamma_{\mathrm{DIC}}$—also known as the Revelle factor(105)—allows for a linear transformation between percentage changes in DIC and pCO_2_, that is, a scaling factor between $\delta DIC/DIC_{\mathrm{ref}}$ and $\delta p{\mathrm{CO}}_{2}/pCO_{2\;\mathrm{ref}}$ (Eq. **2**). Similarly for $\gamma_{\mathrm{Alk}}$ and $\gamma_{\mathrm{SSS}}$. On the other hand, $\gamma_{\mathrm{SST}}$ yields a 4.23% change in pCO_2_ in response to a 1 ^°^C change in SST in absolute terms directly. Differences between the reconstructed $\delta pCO_{2}^{\mathrm{Total}}$ and its simulated value by ICON/HAMOCC are relatively small (*SI Appendix*, Fig. S4*E*), which confirms the robustness of this widely used method.

## Supplementary Material

Appendix 01 (PDF)

## Data Availability

The model code and primary data necessary to reproduce our results are publicly available at the World Data Climate Center (106). Scripts to reproduce the main figures are publicly available from Nielsen et al. (107). All data from the Coupled Model Intercomparison Project phase 6 (CMIP6; 3) are available from the Earth System Grid Federation (ESGF; (108). Satellite observations are publicly available from the National Oceanic and Atmospheric Administration (NOAA) CoastWatch, particularly the chlorophyll-a s DINEOF gap-filling method (70).
