# Supplementary material for: Resolved tropical cyclones trigger CO2 uptake and phytoplankton bloom in an Earth system model simulation
Source: Proc Natl Acad Sci U S A. 2025 Dec 9;122(50):e2506103122. doi: 10.1073/pnas.2506103122 (PMC12718355; doi:10.1073/pnas.2506103122)
Supplement: Supplementary file 1 — Appendix 01 (PDF) [file pnas.2506103122.sapp.pdf]

Supporting Information for

Resolved tropical cyclones trigger CO_2 uptake and phytoplankton bloom in an Earth system model simulation

David Nielsen, Fatemeh Chegini, Nuno Serra, Arjun Kumar, Nils Brüggemann, Cathy Hohenegger and Tatiana Ilyina

Corresponding author: David M. Nielsen
E-mail: david.nielsen@mpimet.mpg.de

This PDF file includes:

Supporting text
Figs. S1 to S15
SI References

Supporting Information Text

A. The phytoplankton-biophysical feedback. It is well understood that increasing near-surface phytoplankton concentrations cause sea-surface warming through the vertical redistribution of heat absorption by chlorophyll at the local scale (1). However, this direct local heating effect may be counterbalanced by indirect dynamical effects. The phytoplankton-driven differential vertical heating causes changes in circulation, which have been shown to have large-scale impacts on sea surface temperature (SST). Manizza et al.(2) showed that the phytoplankton-biophysical feedback cools the surface ocean in the tropics by about 0.3°C due to enhanced upwelling, while it warms SSTs in the subtropics by 0.05°C in their simulations with the ORCA-LIM-PISCES global ocean model. Manizza et al.(3) further showed that changes in large-scale meridional transport also contributes to cooling SSTs in the tropics. On the other hand, Wetzel et al.(4) simulated large-scale tropical sea surface warming in response to the phytoplankton-biophysical feedback in the fully coupled model (ocean-land-atmosphere) MPI-ESM. This apparent discrepancy highlights variability among models, and shows that describing the large-scale impacts of the phytoplankton-biophysical feedback in terms of "SST cooling" vs. "SST warming" is too simplistic. In such studies, one must always assume a fixed profile of light attenuation in a control experiment for comparison, which may vary among models. This limitation and discrepancy is explicitly stated by Wetzel et al.(4) and Manizza et al.(3). Nevertheless, they agree that the mechanism at play is consistent, namely: the modulation of the thermocline by the attenuation depth of shortwave radiation by phytoplankton.

At the local scale, the TC-driven indirect surface cooling effect, caused by secondary changes in circulation, has been suggested to prevail over the direct surface warming in a case study of Hurricane Fernanda (5). In our simulations, we cannot isolate the effect of the phytoplankton-biophysical feedback, which is beyond the scope of our study. In order to do so, we would need a pair of simulations where this feedback is switched on and off for comparison.

B. The role of DIC and alkalinity. The vertical mixing of dissolved inorganic carbon (DIC) and alkalinity play an important role in the impact of TCs on surface pCO_2. The vertical gradients of DIC and alkalinity in the upper ocean are generally similar in the tropical-subtropical ocean, in that concentrations of both quantities increase with depth, especially within the upper ocean (Fig. S6A,B). Consequently, the TC-induced vertical mixing causes an increase in both DIC and alkalinity concentrations in the surface ocean (Fig. S6C,D) with competing effects on surface pCO_2. While the entrainment of high DIC waters increases surface pCO_2, high alkalinity waters decreases surface pCO_2. The net impact of TC-driven changes in surface DIC and alkalinity on surface pCO_2 is small, but predominantly towards an increase in surface pCO_2 in case of TC1 (Fig. S5B).

The sensitivity of surface pCO_2 to DIC and alkalinity is given by linear coefficients γ_{DIC} and γ_{ALK} (see Eq. 2 in Methods). The small net positive change in surface pCO_2 due to changes in DIC+alkalinity (Fig. S5B) can be explained by the magnitude of these coefficients, $|\gamma_{DIC}| \approx 12$ while $|\gamma_{Alk}| \approx 10$ (Fig. S7). That is, a 1% increase in DIC incraeses pCO_2 by ∼12%, while a 1% increase in alkalinity decreases pCO_2 by ∼10% in our simulations in the TC track regions. The relatively stronger role of DIC, compared to that of alkalinity, in modulating surface pCO_2 is in line with observations (6, 7). In addition, γ_{DIC} and γ_{ALK} are spatially uniform (only changing at latitudes >40°N along the TC tracks) and do not change in response to the TCs themselves (Fig. S7). The fact that both γ_{DIC} and γ_{ALK} are insensitive to the TCs underlines that the mechanisms driving changes in surface DIC and alkalinity concentrations in response to TCs are coupled, since both γ_{DIC} and γ_{ALK} are proportional to the DIC-alkalinity ratio (Eq. 3 in Methods). That is, surface DIC and alkalinity increase at the same rate in response to TCs, which keeps their ratio relatively constant. This is visible in the changes in pCO_2 due to changes in DIC and alkalinity individually (Fig. S5F,G), which are strikingly similar but with opposing signs.

The mechanisms driving changes in surface DIC and alkalinity in response to TCs are similar, and overwhelmingly dominated by the vertical diffusion by turbulent mixing (Fig. S8A-D). Biological sinks and sources are the only components of DIC and alkalinity tendencies that have different signals (Fig. S8G,H), but their role is minor compared with the other terms, especially with vertical diffusion (Fig. S8C,D).

C. The impact of biology on surface pCO_2. Changes in biology driven by the passage of the TCs, such as the TC-driven phytoplankton bloom, play a relatively small role in changing surface pCO_2. Any change in surface pCO2 can only be caused by changes in four quantities: dissolved inorganic carbon (DIC), alkalinity, sea surface temperature (SST) and salinity (SSS). Increasing DIC, SST and SSS increases pCO_2, while increasing alkalinity decreases pCO_2. Biological activity affects pCO_2 directly mostly through changes in DIC and alkalinity. Indirectly, phytoplankton also changes the absorption of shortwave radiation, and thus also temperature, as discussed above. However, the impact of biology on surface pCO_2 through temperature is minor compared to that through changes in DIC and alkalinity.

In HAMOCC, the production of 1 mol of organic matter by phytoplankton growth reduces DIC by 122 mols and increases alkalinity by 17 mols, which causes a net reduction in pCO_2. Contrarily, the remineralization of 1 mol of organic matter increases DIC by 122 mols and reduces alkalinity by 17 mols, increasing pCO_2. The ratio between carbon and nutrients in marine organic matter (i.e. the Redfield ratio (8), considered constant in HAMOCC) determines the numbers mentioned above. We express the total change in surface pCO_2 in response to the TCs as the sum of the changes in surface pCO_2 due to DIC, SST, SSS and alkalinity in (Fig. S5D). While changes in SST dominate (Fig. S5A), changes in the pair DIC+alkalinity slightly increase surface pCO_2 in the case of TC1 (Fig. S5B) – not because of biology, but mainly due to the vertical entrainment of DIC due to mixing/diffusion caused by the TC's high wind speeds (Fig. S8C,D) and the higher sensitivity of pCO_2 to DIC than that to alkalinity (Fig. S7). Comparatively, the impact of biology on surface pCO_2 through changing surface DIC or alkalinity is very small (Fig. S8G,H).

D. Organic matter remineralization. The rate of particulate organic matter remineralization R (mol m^{-3} s^{-1}) in HAMOCC increases with temperature T (°C) and oxygen concentrations $[O_2]$ (mol m^{-3}) as detailed by Maerz et al. (9). The dependency of R on temperature is given by a Q10 approach:

$$R = -r \, \frac{[O_2]}{K_{O_2} + [O_2]} \, Q_{10}^{\frac{T - T_{ref}}{10}}$$

where r (mol m^{-3} s^{-1}) is a reference remineralization rate, K_{O_2} (mol m^{-3}) is a half-saturation constant for oxygen, and T_{ref} (°C) is a reference temperature. The rate R, modified by oxygen and temperature, is multiplied by the particulate organic matter concentrations to determine total mass of organic matter remineralization (Fig. 4E in the main text). That is, the amount of organic matter that is remineralized, naturally, also depends on the concentrations of organic matter itself.

In order to isolate the effect of TC-driven temperature changes on remineralization, we computed remineralization rates offline, starting from the model output, but making ocean temperatures constant, fixed to pre-TC conditions. That is, we computed what the remineralization of organic matter would be, if temperature did not change in response to the TCs, but everything else did. Fig. 4F in the main text shows the difference between the fully-resolved remineralization, computed online by HAMOCC (Fig. 4E), and the offline temperature-fixed remineralization. That is, it expresses by how much Fig. 4E would change if temperatures did not change in response to the TCs. The impact of TC-driven changes in temperature on organic matter remineralization is clear: The TC-driven temperature change decreases remineralization in regions where temperature decreases (i.e. pre-TC mixed layer depth), and increases remineralization where temperature increases (i.e. post-TC mixed layer increase). Changes in remineralization due to temperature changes are about 20% in both directions. Vertically integrating these differences (Fig. 4F in the main text) shows that such opposing responses are minor and nearly cancel out each other (red lines), compared to the integrated total remineralization (blue lines). In summary, TC-driven changes in temperature cause as much an increase in remineralization in the shallow pre-TC mixed layer, as a decrease in remineralization below, where the mixed layer becomes deeper. The net effect is small and does not impact organic matter export either at 100 or 1000 meters.

E. Organic matter export. The export of organic matter in HAMOCC is computed as the sinking flux of particulate organic matter, which is dynamically calculated depending on aggregate composition and environmental conditions, according to the parameterization implemented by Maerz et al. (9). Comparing the organic matter export at 90 m with the 0-90 m vertically integrated net primary production (NPP), we see that both curves align well (Fig. S14A). This fact shows that organic matter export increases in response to the TCs, proportionally to the increase in integrated NPP. In addition, we see that the export follows the integrated NPP with a lag of approximately 9 days. Indeed, a lag of some days is expected, since sinking velocities of particulate organic carbon are normally in the order 1-10 meters per day (9). Roughly accounting for a 9-day lag by simply shifting the time series, we see that the simulated TCs do not substantially change the ratio between export at 90 m and the integrated NPP above (i.e. the export ratio). The export ratio in the western North Atlantic ranges at 8-12% throughout the year in our simulations (Fig. S14B).

Direct measurements of organic carbon export in the aftermath of TCs are rare, but there are indications that export increases in response to TCs by between about 20% (10) and up to an order of magnitude (11). In our simulations, we see a maximum increase in export of about 25% between minimum export values in September, before the TCs, and the peak export on October 1st, after the TCs, averaging over the western North Atlantic.

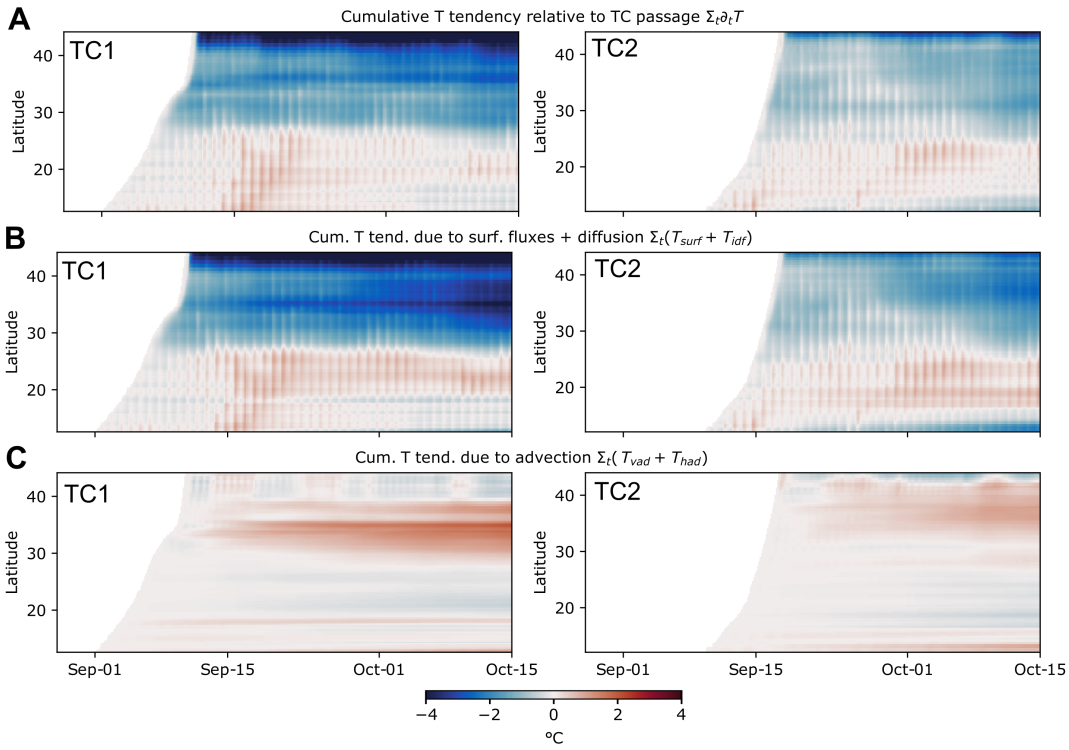

Fig. S1. Changes in sea-surface temperature (SST) relative to pre-TC conditions. Total change in SST **(A)**, expressed as accumulating temperature tendencies from 12 hours before the TC passage, cumulative tendency terms due to the isolated effects of surface heat fluxes and vertical diffusion by turbulent mixing **(B)**, and due to the effects of vertical and horizontal advection **(C)**.

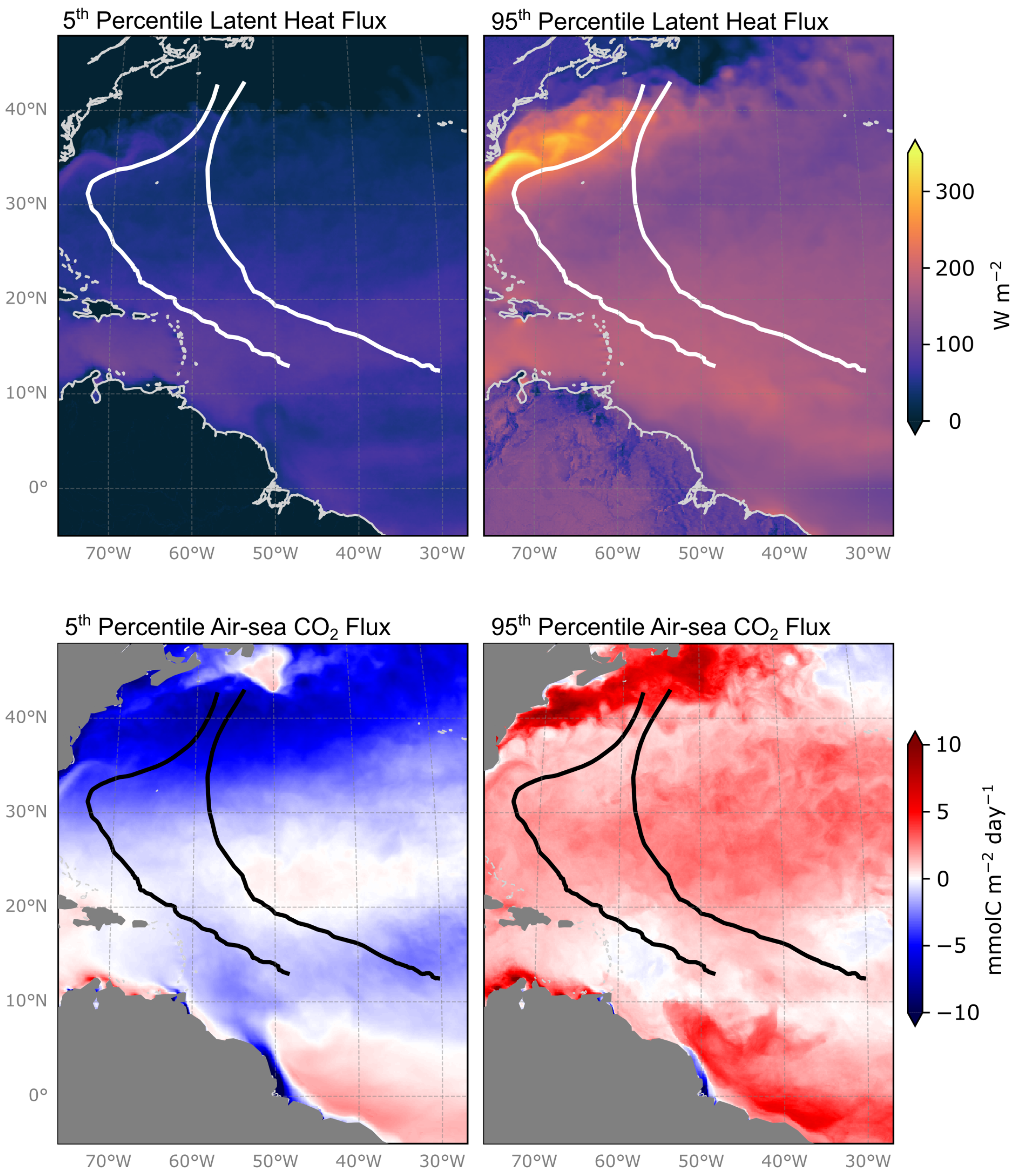

Fig. S2. 5th and 95th percentiles of all-year surface latent heat fluxes (top) air-sea CO_2 fluxes (bottom). Positive values indicate upward fluxes, i.e. from ocean to atmosphere.

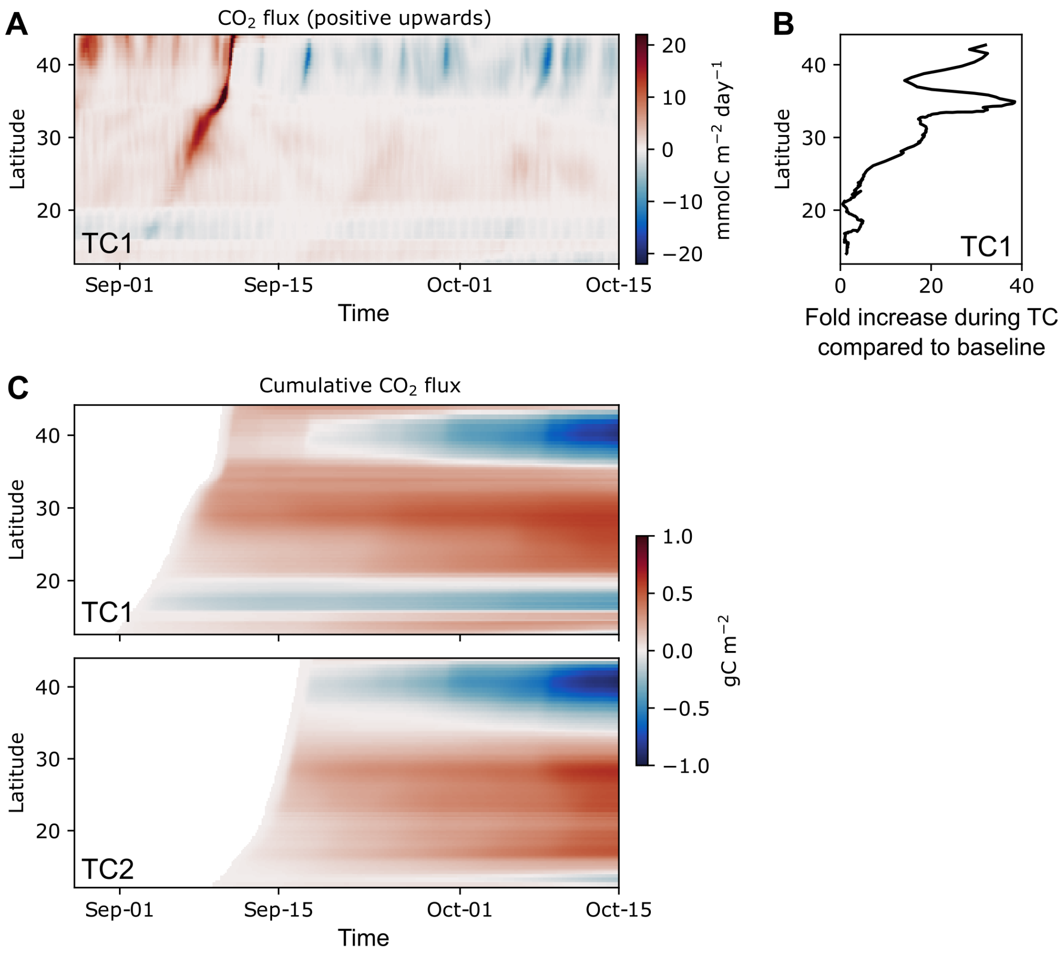

Fig. S3. Impact of TCs on air-sea CO_2 fluxes. Along-track time series of air-sea CO_2 fluxes averaged over a 200-km radius from the TC center (here as in Fig. 2 in the main text, but for TC1) **(A)**, ratio between magnitude of air-sea CO_2 flux during TC1 and baseline, which here is the 1-month period from Oct-1 and Oct-31 excluding the TC days **(B)**, and the cumulative air-sea CO_2 flux starting at 12 hours prior to the TC passage at each location along their tracks **(C)**. A switch from positive to negative cumulative flux in C indicates the moment when the CO_2 uptake following the TC passage outweighs the outgassing triggered during their passage. Units in C are not time dependent because fluxes are integrated temporally.

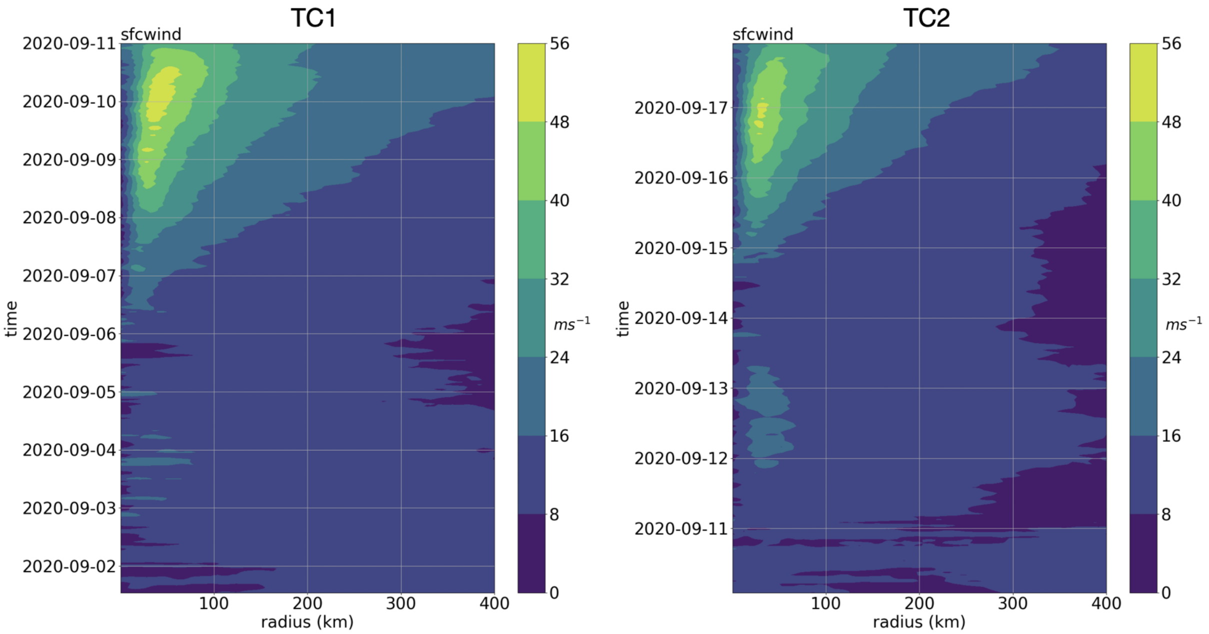

Fig. S4. Temporal evolution of surface wind speeds along the track of TC1 (left) and TC2 (right) as a function of radial distance from cyclone center. We also see that, when the simulated TCs reach hurricane intensity (above 32 m/s), hurricane-intensity wind speeds are found mostly within 200 km from their center. Maximum wind speeds are mostly found within 20-70 km from cyclones' center, which is especially clear at their maximum intensity.

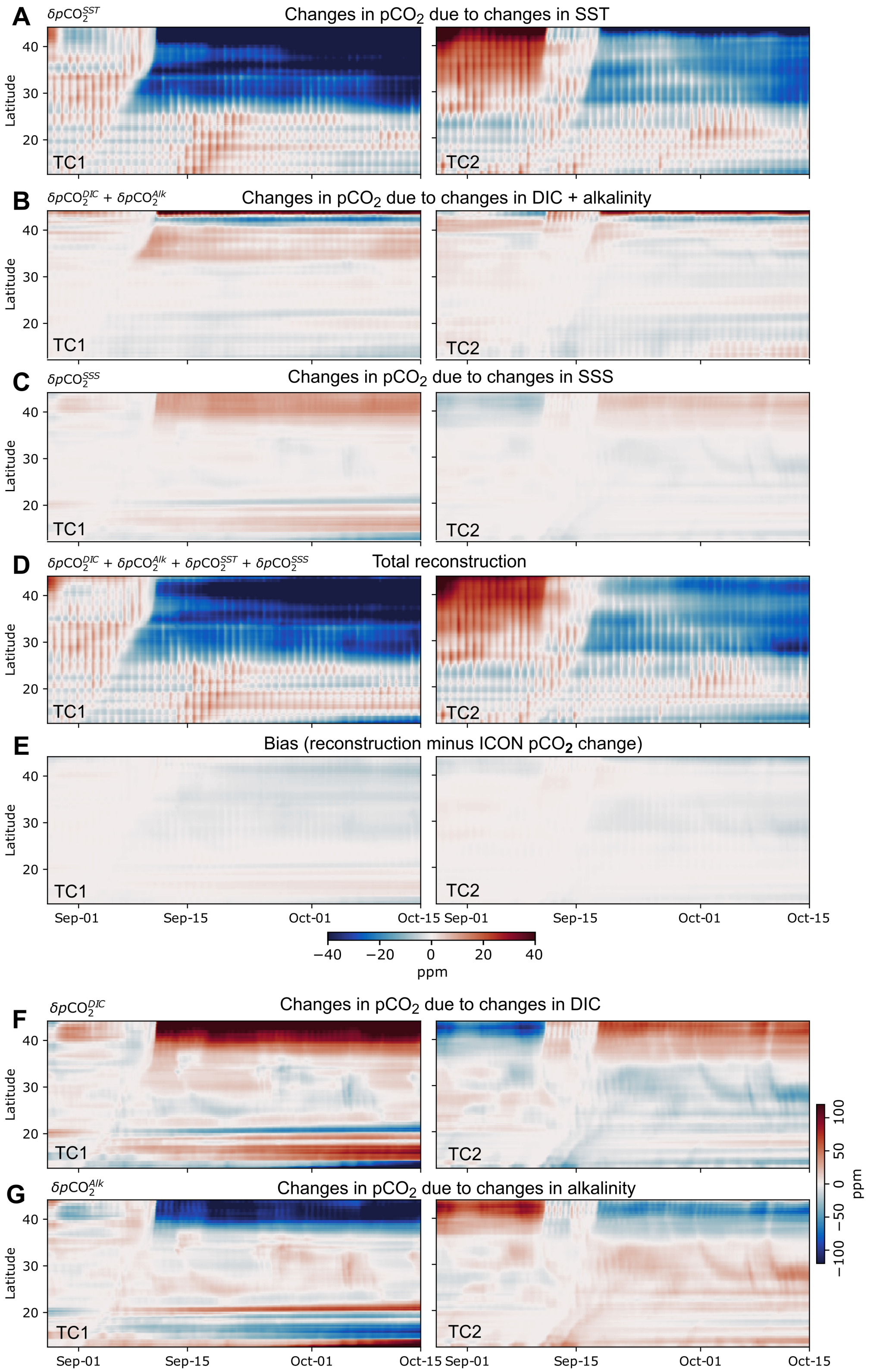

Fig. S5. Along-track time series of changes in surface pCO_2 due to changes in sea-surface temperature (SST, **A**), DIC and alkalinity (**B**), sea-surface salinity (SSS, **C**), their reconstructed combined effect (**D**), and the difference between this reconstruction and the surface pCO_2 changes simulated directly with our model (reconstruction bias, **E**). On the left-hand-side are shown time series along the track of TC1, while on the right-hand-side are shown time series along the track of TC2. We also show the individual impacts of changing DIC (**F**) and alkalinity (**G**) on surface pCO_2 (B=F+G). Note that, in F and G, the limits of the colorbar are extended.

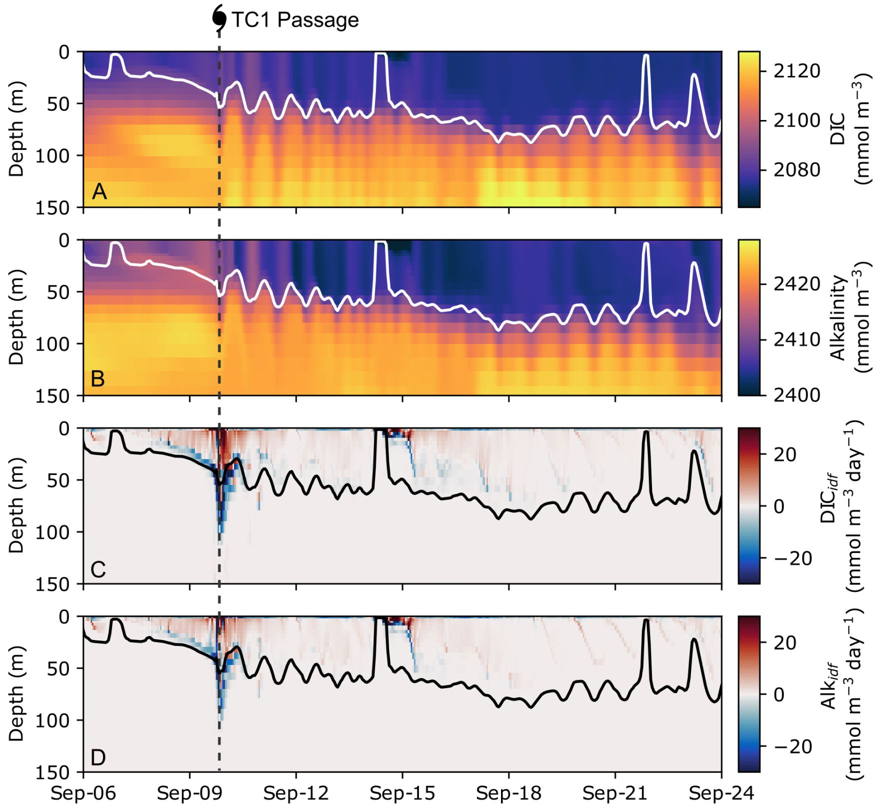

Fig. S6. Time series at approximately 34°N 67°W, on the track of TC2, of vertical sections of dissolved inorganic carbon (DIC, **A**), alkalinity (**B**), and changes in DIC and alkalinity due to vertical diffusion by mixing (**C** and **D** respectively).

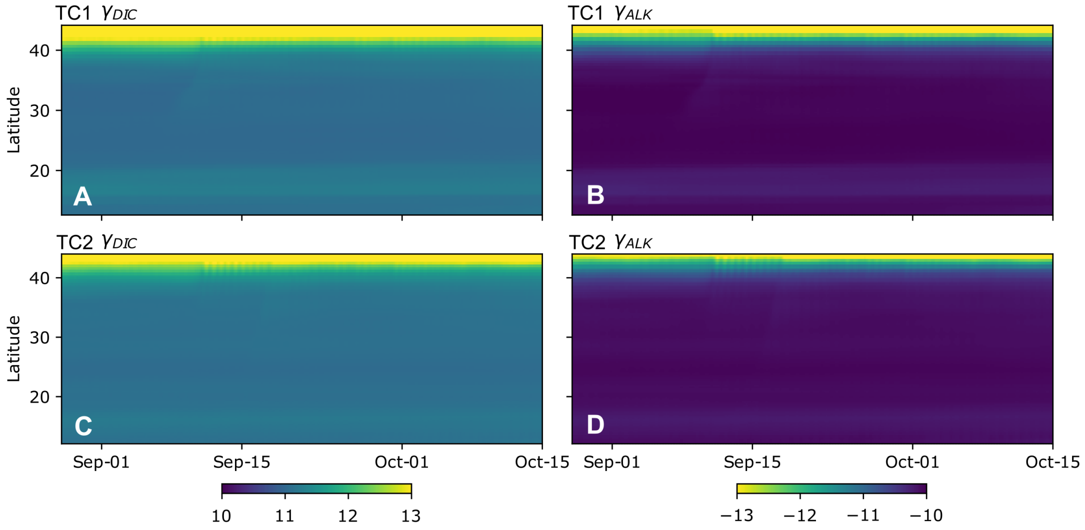

Fig. S7. Along-track time series of changes in the sensitivity coefficients γ_{DIC} and γ_{Alk} in response to TC1 (**A** and **B**, respectively) and in response to TC2 (**C** and **D**, respectively). More details on the sensitivity coefficients are found in the Methods in *'Decomposing changes in pCO₂'*.

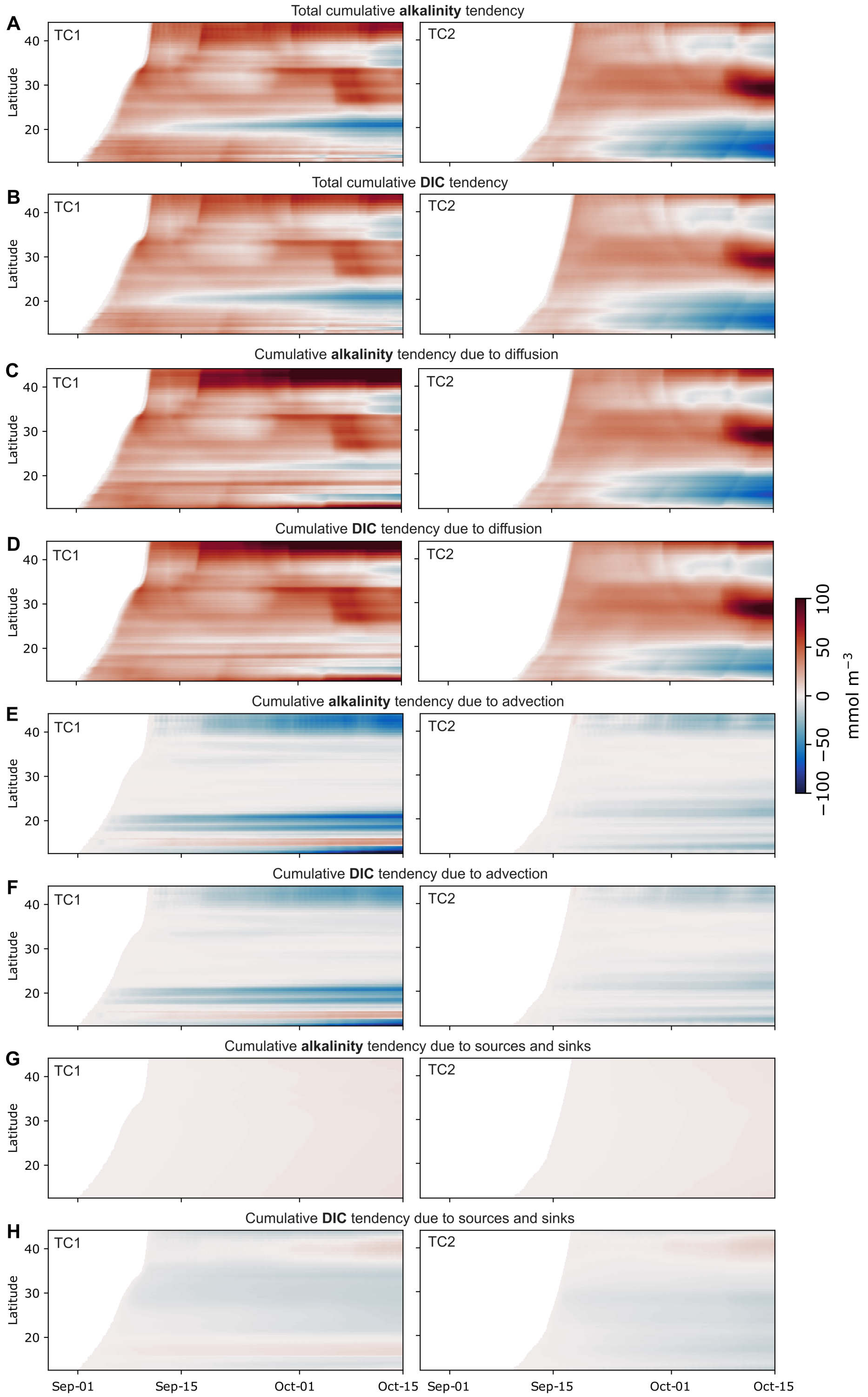

Fig. S8. Along-track time series of changes in surface alkalinity **(A)** and surface DIC **(B)** in response to tropical cyclones expressed as time-cumulative total tendencies, and their components: changes in surface **(C)** and surface DIC **(D)** due to vertical diffusion, changes in surface **(E)** and surface DIC **(F)** due to advection, and changes in surface **(G)** and surface DIC **(H)** due to local biogeochemical sources and sinks. On the left-hand-side are shown time series along the track of TC1, while on the right-hand-side are shown time series along the track of TC2. Units are mmol/m^3 because tendency terms are integrated (or accumulated) in the time dimension, and therefore they express changes in the concentrations themselves.

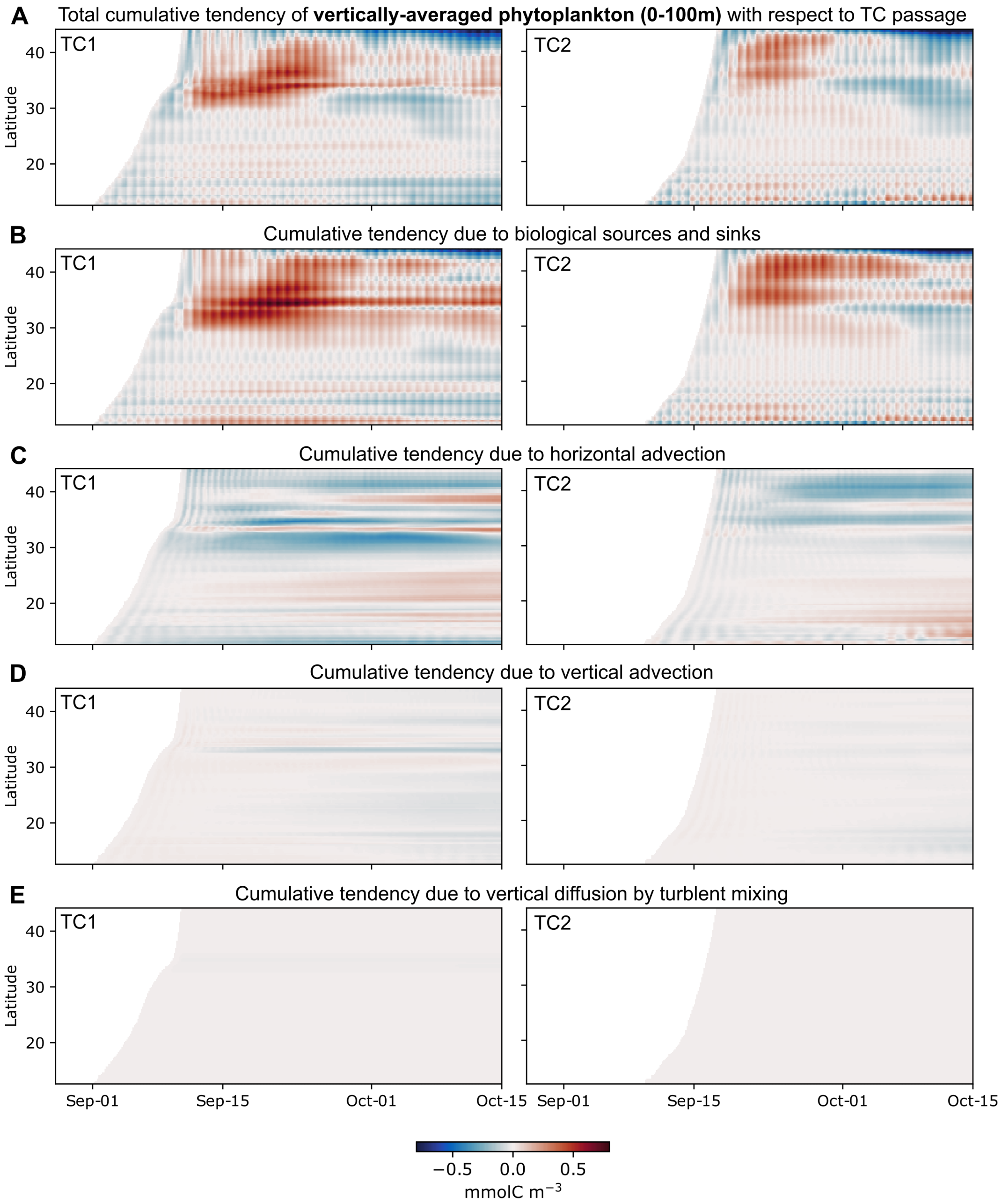

Fig. S9. Along-track time series of cumulative tendencies of integrated (0-100 m) phytoplankton concentrations **(A)** and its components, that is, changes in phytoplankton concentrations due to biological sources and sinks **(B)**, horizontal advection **(C)**, vertical advection **(D)**, and wind-driven vertical diffusion by mixing **(E)**. Units do not depend on time because tendency terms are integrated (or accumulated) in the time dimension. Accumulating periods start 12 hours before the TCs passage.

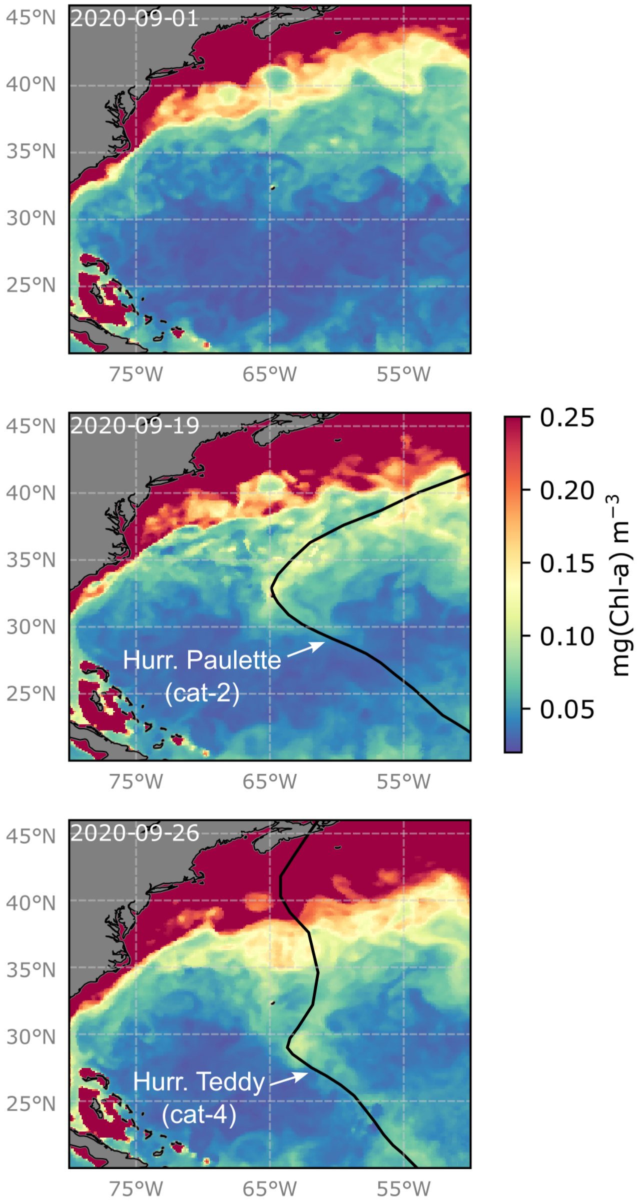

Fig. S10. Satellite-derived Chlorophyll-a observations on 01-09-2020, 19-09-2020, and 26-09-2020, overlaid with the tracks of TCs Paulette and Teddy. TC Paulette reached peak intensity to hurricane category 2 on 10-09-2020 , and made landfall in Bermuda on 14-09-2020. TC Teddy reached peak intensity on 17-09-2020 to hurricane category 4. Black lines show the TC tracks. Chlorophyll-a data are obtained from (12), and TC tracks are obtained from NOAA's Atlantic hurricane database (HURDAT2) (13).

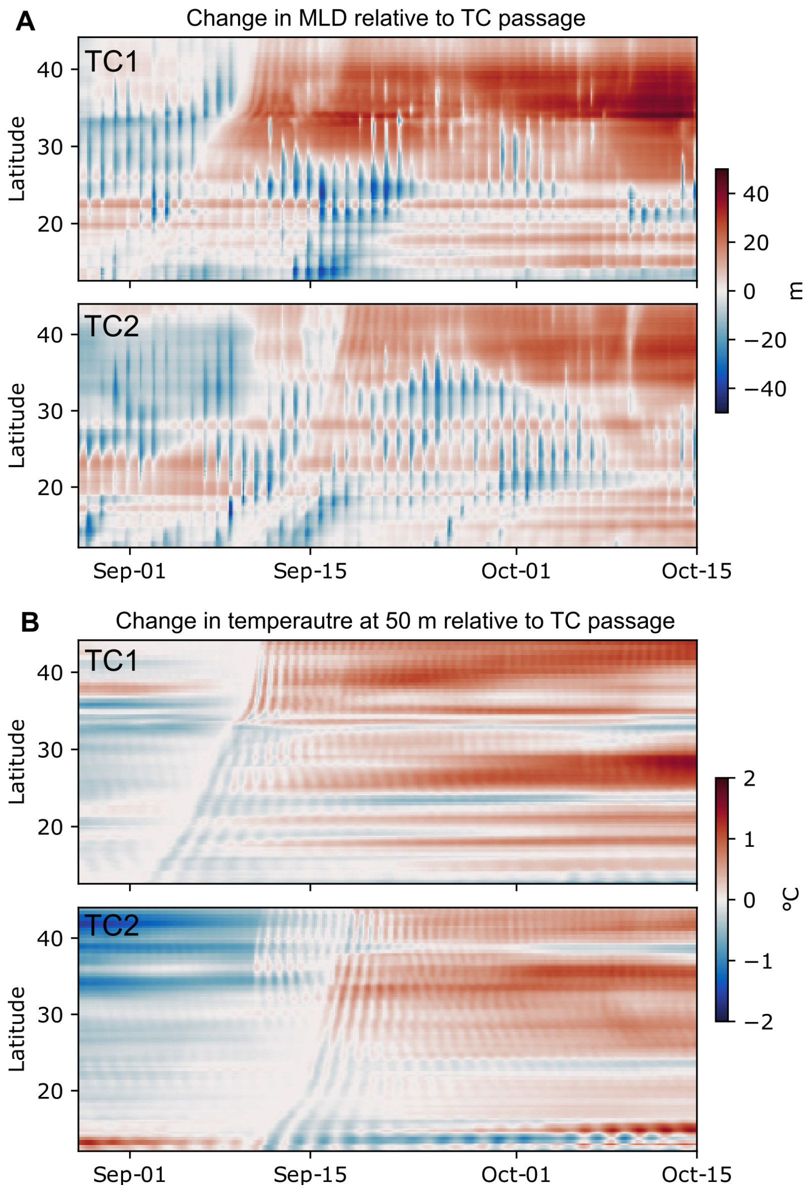

Fig. S11. Along-track time series of changes in mixed-layer depth (MLD, **A**) and in temperature at 50 m (**B**) with respect to the passage of TCs.

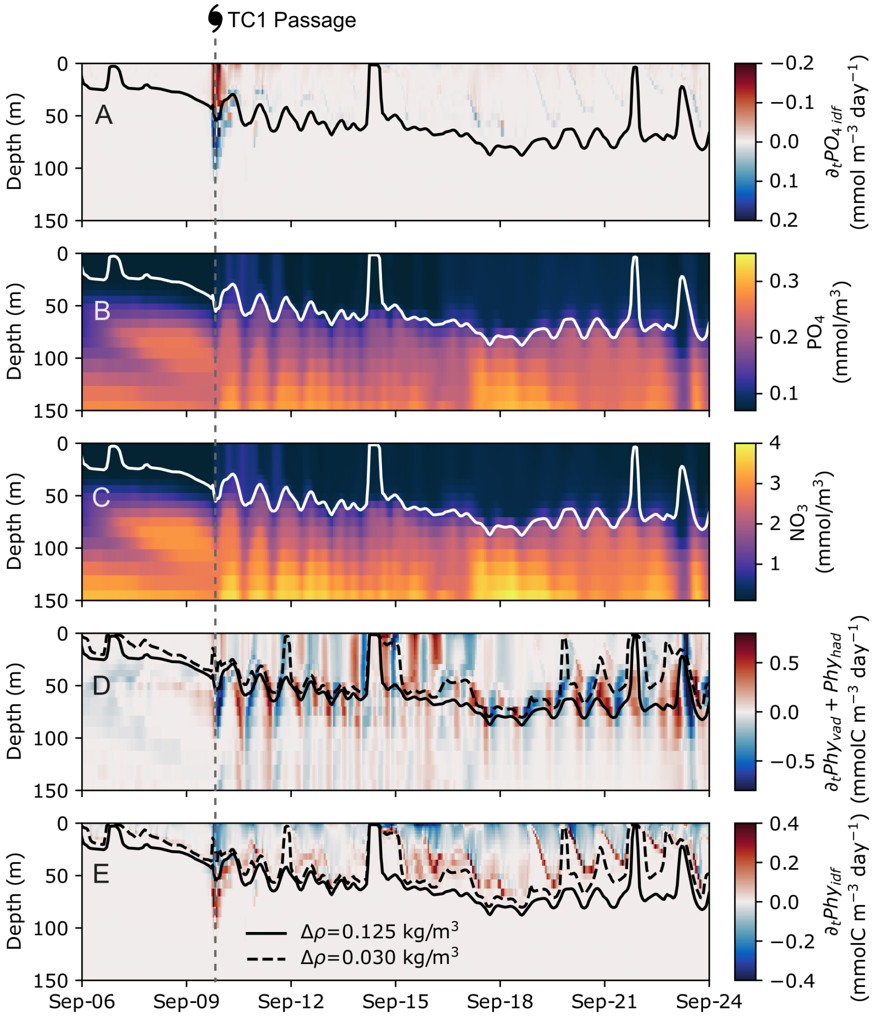

Fig. S12. Time series at 37°N 67°W, continuation of Fig. 4 in the main text. Here shown are changes in phosphate concentrations due to vertical diffusion by wind-driven mixing **(A)**, phosphate concentrations **(B)**, changes in phytoplankton concentration due to horizontal and vertical advection **(C)** and due to vertical diffusion by mixing **(D)**

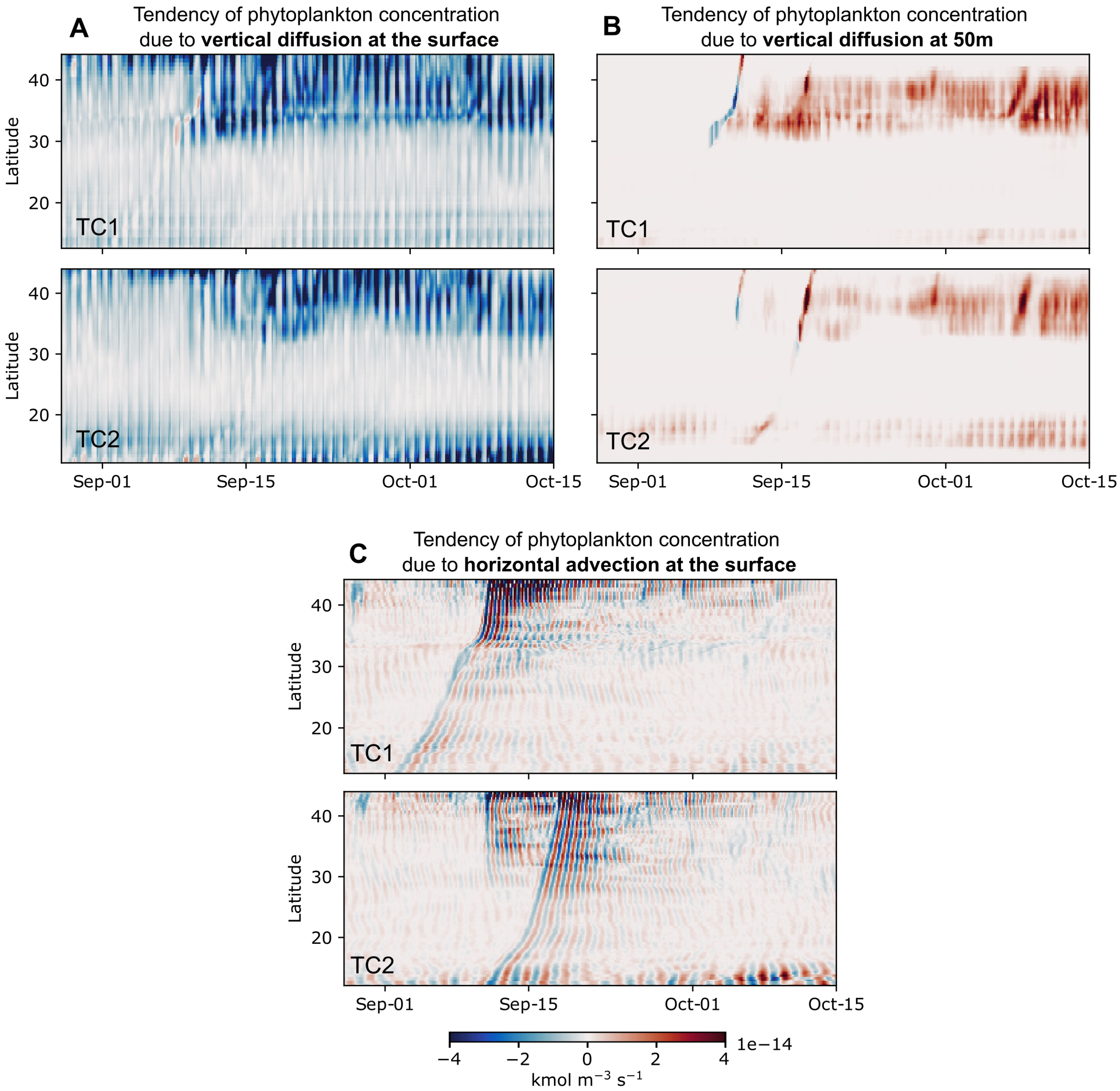

Fig. S13. Along-track time series of changes in phytoplankton concentration due to horizontal advection and vertical diffusion. Quantities are averaged from a 150 km-radius circle following the center of the TCs along their tracks. The left vertical axes show the along-track distance from the first point where the TC is detected. The right vertical axes show the latitude coordinate of the center of TCs along their tracks. The vertical advection components of phytoplankton tendency show a similar pattern to those of horizontal advection, but with opposite sign, due to continuity. Note the dominant role of inertial oscillations, and their increasing frequency with latitude, in the variability of the advection terms. Data are *not* filtered.

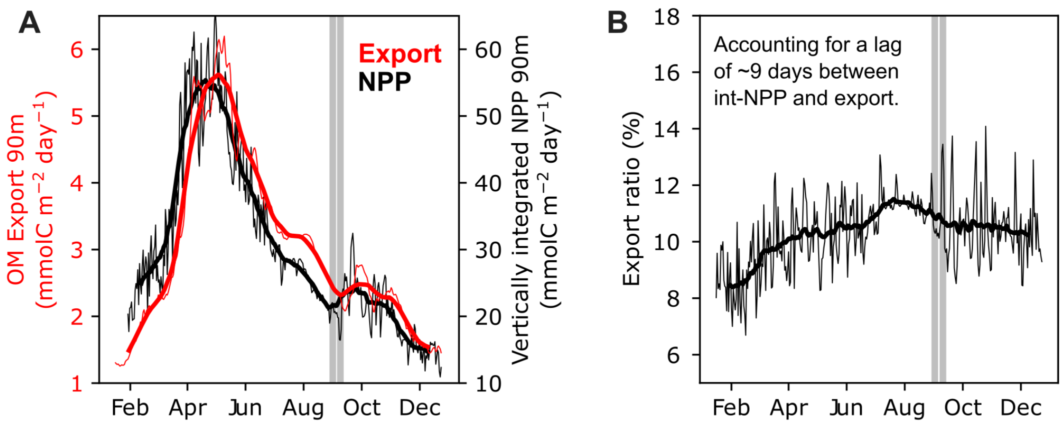

Fig. S14. Seasonal cycle of organic matter export at 90m and vertically-integrated net primary productino (NPP) unil 90 m (**A**), and the ratio between organic matter export at 90 m and the integarted NPP expressed as percentages, accounting for a 9-day lag (export after NPP, **B**). All time series are spatially-averaged over the western North Atlantic (triangular region in Fig. 3A). Thin lines show daily means, and thick lines show 31-day running means of daily means. The passage of the simualted TCs are marked by the grey vertical bars.

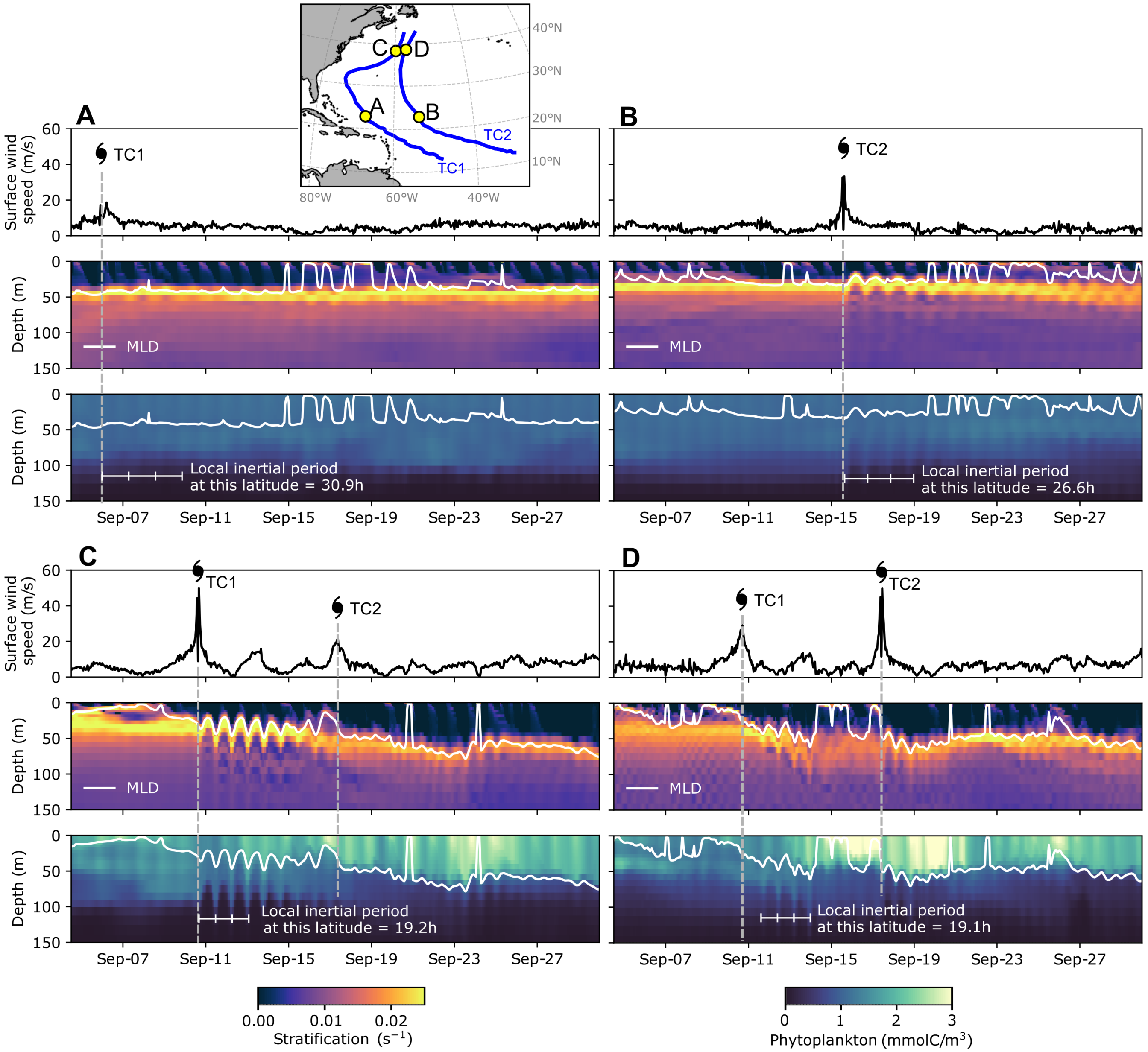

Fig. S15. Time series of surface wind speed, vertical density stratification, and phytoplankton concentration at selected points, **A:** 22.8°N 67.1°W, **B:** 22.7°N 57.0°W, **C:** 38.5°N 59.5°W, and **D:** 38.9°N 56.8°W. Points A and C are on the trajectory of TC1, while points B and D are on the trajectory of TC2 (see map). The moment TCs cross the selected points are indicated by vertical dashed lines. White contours indicate the mixed layer depth, and horizontal white axes illustrate the inertial period at the respective latitudes.

References

1. A Morel, D Antoine, Heating rate within the upper ocean in relation to its bio–optical state. *J. Phys. Oceanogr.* **24**, 1652 – 1665 (1994).
2. M Manizza, C Le Quéré, AJ Watson, ET Buitenhuis, Bio-optical feedbacks among phytoplankton, upper ocean physics and sea-ice in a global model. *Geophys. Res. Lett.* **32**, GL020778 (2005).
3. M Manizza, C Le Quéré, AJ Watson, ET Buitenhuis, Ocean biogeochemical response to phytoplankton-light feedback in a global model. *J. Geophys. Res. Ocean.* **113**, C10010 (2008).
4. P Wetzel, et al., Effects of ocean biology on the penetrative radiation in a coupled climate model. *J. Clim.* **19**, 3973 – 3987 (2006).
5. S Ye, RH Zhang, H Wang, F Tian, Q Shi, Tropical cyclone-induced ecological responses and their feedback on physical fields: A case study for hurricane fernanda (2017). *J. Geophys. Res. Ocean.* **129**, e2024JC021150 (2024).
6. T Takahashi, J Olafsson, JG Goddard, DW Chipman, SC Sutherland, Seasonal variation of co_2 and nutrients in the high-latitude surface oceans: A comparative study. *Glob. Biogeochem. Cycles* **7**, 843–878 (1993).
7. JL Sarmiento, N Gruber, *Ocean Biogeochemical Dynamics.* (Princeton University Press), (2006).
8. A Redfield, On the proportions of organic derivatives in seawater and their relation to the composition of plankton. *James Johnstone Meml. Vol.* pp. 176–192 (1934).
9. J Maerz, KD Six, I Stemmler, S Ahmerkamp, T Ilyina, Microstructure and composition of marine aggregates as co-determinants for vertical particulate organic carbon transfer in the global ocean. *Biogeosciences* **17**, 1765–1803 (2020).
10. KS Chen, et al., Enhanced POC export in the oligotrophic northwest pacific ocean after extreme weather events. *Geophys. Res. Lett.* **40**, 5728–5734 (2013).
11. R Pedrosa-Pàmies, MH Conte, JC Weber, R Johnson, Hurricanes enhance labile carbon export to the deep ocean. *Geophys. Res. Lett.* **46**, 10484–10494 (2019).
12. X Liu, M Wang, Global daily gap-free ocean color products from multi-satellite measurements. *Int. J. Appl. Earth Obs. Geoinformation* **108**, 102714 (2022).
13. CW Landsea, JL Franklin, Atlantic hurricane database uncertainty and presentation of a new database format. *Mon. Weather. Rev.* **141**, 3576 – 3592 (2013).
